# Data-driven crop growth simulation on time-varying generated images using multi-conditional generative adversarial networks

**DOI:** 10.1186/s13007-024-01205-3

**Published:** 2024-06-15

**Authors:** Lukas Drees, Dereje T. Demie, Madhuri R. Paul, Johannes Leonhardt, Sabine J. Seidel, Thomas F. Döring, Ribana Roscher

**Affiliations:** 1 Remote Sensing Group, Institute of Geodesy and Geoinformation, University of Bonn, Niebuhrstr. 1a, Bonn, 53113 Germany; 2 Crop Science Group, Institute of Crop Science and Resource Conservation, University of Bonn, Katzenburgweg 5, Bonn, 53115 Germany; 3 Agroecology and Organic Farming Group, Institute of Crop Science and Resource Conservation, University of Bonn, Auf Dem Hügel 6, Bonn, 53121 Germany; 4 Data Science For Crop Systems, Forschungszentrum Jülich GmbH, Wilhelm-Johnen-Straße, Jülich, 52428 Germany

**Keywords:** Machine learning, Image generation, Conditional GAN, Growth modeling, Crop mixtures

## Abstract

**Background:**

Image-based crop growth modeling can substantially contribute to precision agriculture by revealing spatial crop development over time, which allows an early and location-specific estimation of relevant future plant traits, such as leaf area or biomass. A prerequisite for realistic and sharp crop image generation is the integration of multiple growth-influencing conditions in a model, such as an image of an initial growth stage, the associated growth time, and further information about the field treatment. While image-based models provide more flexibility for crop growth modeling than process-based models, there is still a significant research gap in the comprehensive integration of various growth-influencing conditions. Further exploration and investigation are needed to address this gap.

**Methods:**

We present a two-stage framework consisting first of an image generation model and second of a growth estimation model, independently trained. The image ﻿generation model is a conditional Wasserstein generative adversarial network (CWGAN). In the generator of this model, conditional batch normalization (CBN) is used to integrate conditions of different types along with the input image. This allows the model to generate time-varying artificial images dependent on multiple influencing factors. These images are used by the second part of the framework for plant phenotyping by deriving plant-specific traits and comparing them with those of non-artificial (real) reference images. In addition, image quality is evaluated using multi-scale structural similarity (MS-SSIM), learned perceptual image patch similarity (LPIPS), and Fréchet inception distance (FID). During inference, the framework allows image generation for any combination of conditions used in training; we call this generation data-driven crop growth simulation.

**Results:**

Experiments are performed on three datasets of different complexity. These datasets include the laboratory plant *Arabidopsis thaliana* (Arabidopsis) and crops grown under real field conditions, namely cauliflower (GrowliFlower) and crop mixtures consisting of faba bean and spring wheat (MixedCrop). In all cases, the framework allows realistic, sharp image ﻿generations with a slight loss of quality from short-term to long-term predictions. For MixedCrop grown under varying treatments (different cultivars, sowing densities), the results show that adding these treatment information increases the generation quality and phenotyping accuracy measured by the estimated biomass. Simulations of varying growth-influencing conditions performed with the trained framework provide valuable insights into how such factors relate to crop appearances, which is particularly useful in complex, less explored crop mixture systems. Further results show that adding process-based simulated biomass as a condition increases the accuracy of the derived phenotypic traits from the predicted images. This demonstrates the potential of our framework to serve as an interface between a data-driven and a process-based crop growth model.

**Conclusion:**

The realistic ﻿generation and simulation  of future plant appearances is adequately feasible by multi-conditional CWGAN. The presented framework complements process-based models and overcomes their limitations, such as the reliance on assumptions and the low exact field-localization specificity, by realistic visualizations of the spatial crop development that directly lead to a high explainability of the model predictions.

## Background

Growing crops sustainably, i.e., producing sufficient agricultural output with high resource use efficiency and minimal negative impacts on ecosystems, requires complex optimization of crop management [1]. Decisions on the operations during the season include the timing and amounts of fertilization, irrigation, protection against pests and pathogens, weeding, applying growth regulations, and other activities. The optimality of most of these operations and their combinations depend on the phenology of crops, i.e., the growth stages and size of the plants. Complex and multiple interactions typically occur between different management factors, crop genotypes, and variable environmental factors, affecting crop performance differently at different growth stages. Because of this complexity, identifying optimized crop management is not trivial, and various ways have been developed to tackle this problem and to understand crop responses to complex management $\times$ environment interactions. Two complementary approaches are experimental field trials and process-based (mechanistic) crop growth monitoring. While field experimentation integrates actual environmental and management conditions, it is limited in time and space and can only test a low number of such conditions. Crop growth modeling, on the other hand, while allowing the simulation of multiple conditions, including future environments, is always a simplification of the situation in the field and may be limited in predicting realistic responses of crops, especially under a changing climate [2]. Because of the central role of crop phenology in agronomic decision-making, it is useful to predict future crop growth stages and crop appearance in the season. One pathway towards this goal is the automatic generation of crop images derived from images taken during earlier stages. This is particularly difficult but also useful in crop mixtures, where interactions occur between two or more crop species grown together on the same field.

As an example, cereal and legume crop mixtures are known to improve resource use efficiency [3], enhance nutrient acquisition [4], maximize system productivity through complementarity, especially on low input land limited by nitrogen deficiency [5], and reduce weeds, diseases, and insect pest infestations [6]. Nevertheless, many farmers do not consider crop mixtures as an option, often due to a knowledge gap in species, cultivar, and treatment selection, which results in performance uncertainty [7]. Predictive crop modeling is one approach to dealing with complexity and overcoming this uncertainty.

The differences between predictive crop growth models are manifold. Process-based models are based on biological and physical relationships and aim to represent the mechanics of plant growth and thus have a high interpretability. They are also suitable for long-term predictions and can be generalized to different locations, but both require a complex calibration to the respective environment. Image-based crop growth models, on the other hand, are data-driven, with information on the actual crop environment encoded in the image. By using machine learning to process this image data, data-driven models can build complex relationships [8] without relying on simplified assumptions, like process-based models. The modeling process becomes less interpretable, but the resulting predicted image, depicting a realistic future spatial plant development and derived phenotypic traits, can be more effectively explained in a human-understandable manner. The predicted image is highly versatile, which is particularly interesting for crop mixtures, e.g., to count the future number of crops at certain field positions or to visualize, and thus better understand, how two species behave and compete with respect to certain influencing factors. Therefore, this work aims to extract more insight from image-based models to complement missing facets of existing well-established crop growth models.

In recent years, the most widely used method for image generation in plant science has been Generative Adversarial Networks (GANs) [9], as they have proven to generate high-quality images. In particular, its conditional GAN (CGAN) variant has found a wide application, e.g., to generate realistic plant images [10–12] for data augmentation, or segmentation [13]. While these works operate in the same temporal domain, few works exist that incorporate the factor of time to generate and analyze probable future growth stages. Yasrab et al. [14] generate segmentation images of future root and shoot systems of Arabidopsis (*Arabidopsis thaliana*) and Komatsuna (*Brassica rapa*) based on a time series of past images. However, their GAN model is limited to observation times with fixed constant intervals, severely limiting the space of possible input time series and making long-term ﻿predictions difficult. Furthermore, due to significant differences in the bit depth, the generation of segmentations is much less complex than the generation of artificial plant images, which can be considered as artificial sensor data. Drees et al. [15] show long-term predictions of realistic images of the above-ground plant phenotype. However, it has the disadvantage that time is not explicitly included as a condition, so the image generation is limited to predefined growth prediction steps between fixed growth stages. This challenge can be addressed by extending the generator with modules responsible for integrating the time factor, such as a combination of positional encoded time points and a transformer encoder, as shown in [16]. This allows the flexible integration of multiple time points as a condition and the generation of an arbitrary growth stage in the output. However, the image quality is not optimal because the model is limited to a small bottleneck dimension due to a parameter-intensive Transformer encoder. Further, the evaluation in this work is based only on classical metrics, such as structural similarity, but lacks crucial plant-specific evaluations that demonstrate actual usability by deriving phenotypic traits. In general, all the methods mentioned above have the disadvantage that plant growth is greatly simplified by considering only other growth stages, so the time factor in the input, while it is subject to complex mechanisms. Miranda et al. [17] attempt to get closer to this complexity by integrating more conditions into the growth modeling, which allows them to generate controlled and more explainable output images. However, the method is limited to continuous conditions and a predefined growth prediction step from a fixed early growth stage to a fixed later growth stage, which is unfavorable in agricultural practice. Integrating multiple conditions is generally a non-trivial task, as conditioned image generation tends to generate deterministic and less diverse outputs up to mode collapse [18]. There are many different ways of integrating conditions from concatenation [19] over auxiliary classifiers [20] and latent projection [21] to conditional batch normalization [22, 23]. This work uses the latter because it allows the intuitive integration of multiple conditions while maintaining the stochasticity of the model to create an adequate distribution of generated plants.

**Fig. 1:**
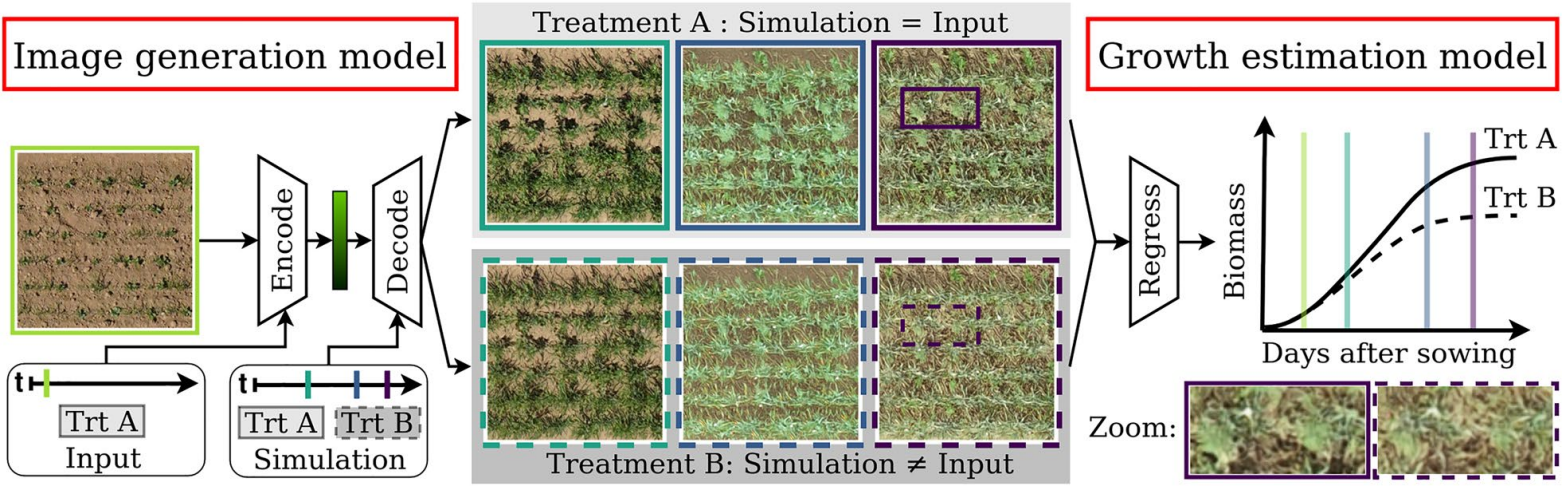
Proposed two-step crop growth simulation framework: In the first step of image ﻿generation, an input image is initially encoded with its associated time (t) and treatment (trt). Then, this encoded representation can be decoded into newly generated images with varying growth stages for different simulation times and treatments (gray boxes). In the second step of growth estimation, target traits such as projected leaf area or biomass are estimated from the images and analyzed over time. Both models are trained independently

An overview of our growth simulation framework is depicted in Fig. 1. It is a two-step procedure in which time-varying images are first generated with the image generation model and then analyzed with an independently trained growth estimation model. An important novelty in the image generation model, which is a Conditional Wasserstein GAN (CWGAN), is the integration of multiple conditions of different types, that is, images (2D spatial continuous variables), time points (discrete), treatment information (categorical), and daily simulated biomass (continuous). Since the biomass is process-based simulated, we demonstrate that the image generation model can serve as an interface that makes the output of process-based crop growth models more explainable by visualizing the spatial crop development. Conditioning is realized by conditional batch normalization in both parts, the encoder and decoder, of the CWGAN generator. This enables simulations during inference, i.e., while fixing initial conditions (input image, time, and treatment), for other growth stages, conditions can be varied—as required—to generate multiple realistic predictions, as shown in Fig. 1. Experiments have been conducted on different datasets of varying complexity, from the plant *Arabidopsis thaliana* (Arabidopsis) to real field data with cauliflower (GrowliFlower) and crop mixtures (MixedCrop). In addition to classical GAN evaluation measures, we evaluate the quality of generated images through the growth estimation model, which acts as a plant phenotyping module, by comparing (depending on the dataset) the projected leaf area or the biomass estimated from generated and real images. For crop mixtures, this allows us to compare our image-based crop growth model and a classic process-based one with biomass-cutting references obtained in the field. A transferability experiment demonstrates that our framework has the potential to be transferred to crop mixtures in another field with different environmental conditions.

## Materials and methods

This section introduces the data basis (see Data section) and the framework,^1^ where a 2-step approach is followed. First, an image is generated (see Image generation section), and second, the growth is estimated using plant phenotyping (see  Growth estimation section). While existing state-of-the-art models are used for growth estimation, which is fine-tuned on our data, the methodological focus of this work is on the first part, image generation. We also provide details about the process-based crop growth model (see Process-based modeling of crop mixtures section) used to evaluate and analyze generated images of crop mixtures.

To specify the terminology: We call the output of the image generation model generated or predicted image. The output of the whole framework is called data-driven prediction, in contrast to the process-based prediction, which is the process-based simulated biomass. In the case of predictions, there is always a time shift $\Delta t=t_{\text {gen}}-t_{\text {in}}$, so $\Delta t>0$ means prediction into the future, $\Delta t<0$ means prediction into the past, and Δt=0 is an identity mapping.  The output of the growth estimation model is an estimation (no time shift) relating to its own input but a prediction relating to the input of the preceding image generation model.

### Data

**Table 1:** Dataset characteristics

	Arabidopsis	GrowliFlower	MixedCrop	
			Mixed-CKA	Mixed-WG
# images	54,384	102,264	21,371	18,800
Observation period [d]	18	71	113	109
# times^1^ (Cond.: **t**)	850	12	11	10
# sequences^2^	64	8522	2226	2212
# train sequences	40	6572	1555	1580
# val sequences	8	979	311	316
# test sequences	16	971	311	316
$\varnothing$ images/sequence	850	12	9.60	8.50
image size [px]	256	256	256	256
GSD [mm]	0.23	3.10	5.67	5.67
diff. treatments (Cond.: **trt**)	$\times$	$\times$	$\checkmark$ (76)	$\checkmark$ (76)
sim. biomass (Cond.: **bm**)	$\times$	$\times$	$\checkmark$	$\checkmark$

GEM: # train images	512	1541	15,017	13,154
GEM: # val images	148	326	3177	2823
GEM: # test images	148	330	3177	2823

The upper block indicates the image specifications for the image generation model, where the different conditions time (t), treatment (trt), and biomass (bm) are highlighted, and the bottom block displays the number of images used to train, validate, and test the resp. growth estimation model (GEM), which is trained independently on individual images without sequence information.

^1^ The number of different time points equals the max. sequence length; for Arabidopsis, it is greater than the period because up to four images were taken per hour

^2^ The number of sequences equals the number of different plants in Arabidopsis and spatially separated field patches in GrowliFlower and MixedCrop

**Fig. 2:**
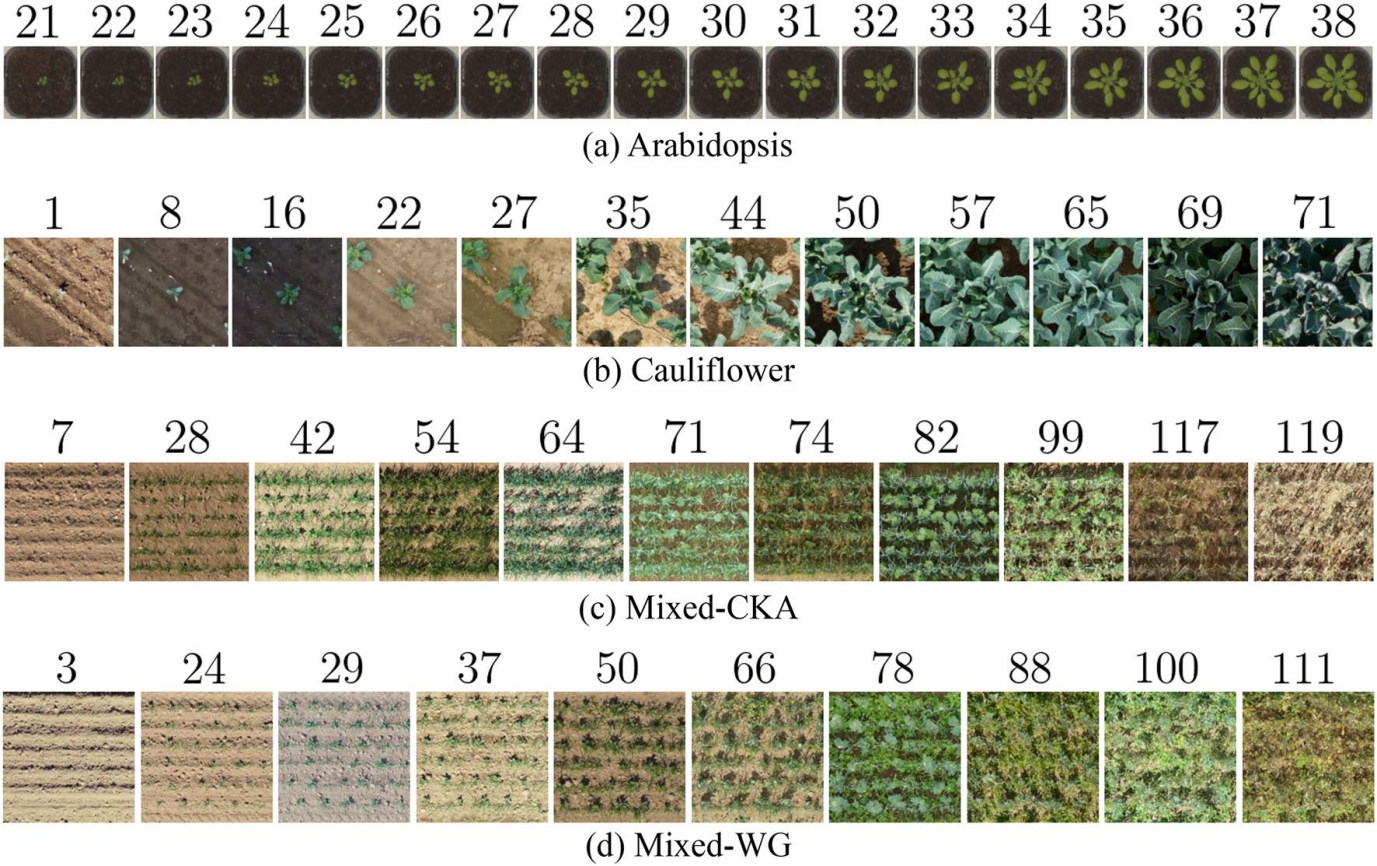
Example evolution over time of one plant resp. from each of the datasets (**a**) Arabidopsis, (**b**) GrowliFlower, (**c**) Mixed-CKA, and (**d**) Mixed-WG visualized by georeferenced clips from RGB orthophotos. The number above the images indicates the growth stage for (**a**), (**c**) and (**d**) in days after sowing [DAS] and for (**b**) in days after planting [DAP]

Experiments are set up on three different datasets: Arabidopsis, GrowliFlower, and MixedCrop, all containing RGB-image time series/sequences of plants (Fig. 2). They meet the minimum requirement of having aligned images, meaning that all images of a sequence show the same region from the same perspective and resolution over time. Ideally, lightning conditions are constant, which is only the case for the Arabidopsis dataset. Beyond that, they differ in essential aspects such as overall size, type of plants, heterogeneity of images, and number, as well as regularity of acquisition times during the vegetation period. Notably, additional conditions on treatment and daily simulated biomass are available only for MixedCrop, all listed in Tab. 1.

*Arabidopsis* The Arabidopsis dataset [24] includes 80 different Arabidopsis (*Arabidopsis thaliana*) plants recorded on four trays of 20 plants each over a 35d period using an IDS UI-5480SE camera (Tamron 8mm f1.4 lens, 5MP). The camera was mounted on a robotic arm in a controlled laboratory environment, ensuring image alignment. All tray images are corrected for barrel distortions with a provided calibration script [24] and then manually cropped at the edges of the pots, resulting in images with a single plant in the center region. We focus on images from 18 days of early developmental stages of Arabidopsis thaliana from day 21 after sowing, which is shortly after plant emergence, to day 38 after sowing. Any plants removed from the experiment before day 38 or protruding beyond the pot’s edge after day 38 were filtered out, leaving 64 plants. Please note that the number of images per sequence exceeds the duration of the observation period in [d] because not only one image per day was taken, but up to four per hour.

*GrowliFlower* The GrowliFlower dataset [25] contains a total of 102 264 images of cauliflower (*Brassica oleracea var. botrytis*) in 2021 from a field in Bornheim, Rhein-Sieg Kreis, Germany. We use images within the 71-day period after planting and exclude images after harvest. The images are orthophoto crops taken from a drone equipped with a Sony Alpha 7R III camera (Zeiss/Batis 2.0 lens, ${47.4}\,\hbox {MP}$). The geo-referencing of the orthophotos allows aligned plant-centered cropping at the same position at each point in time. However, compared to Arabidopsis, there is not only one plant per image, but more heads are visible at the image edges and overlap in later growth stages.

*MixedCrop* The MixedCrop data are from a 2020 and 2021 PhenoRob crop mixture experiment described in detail by Paul et al. [26]. Two different cultivars of faba bean (FB, *Vicia faba*) and twelve different entries of spring wheat (SW, *Triticum aestivum*) were sown in mixtures of a 1:1 ratio, which means 50 % of seeds of each species from the respective monoculture as well as in monocultures. The field experiments were conducted at two research sites of the University of Bonn in the Rhein-Sieg-Kreis, Germany, namely, Campus Klein-Altendorf (CKA, near Rheinbach) and at Wiesengut (WG, near Hennef). Coupled with two different seeding densities i.e. low (L) 80% and high (H) 120% of the recommended sole crop densities (400 seeds $\text {m}^{-2}$ for SW and 45 seeds $\text {m}^{-2}$ for FB), this results in $(2\cdot 12+2+12)\cdot 2=76$ different treatments, which were replicated four times, or, in case of the faba bean monocultures, eight times, resulting in a total of 320 different plots of size ${10}\,\hbox {m}\times {1.5}\,\hbox {m}$ at each of the two sites. Both experimental sites are located about 30 km apart and have significantly different growing conditions because Mixed-CKA is managed conventionally and Mixed-WG organically.

The image acquisition was conducted in 2020—11 times for Mixed-CKA and 10 times for Mixed-WG by UAV equipped with an FC6310 camera (1” CMOS 8.8mm, 20MP). The 320 field plots are positional-aligned cropped from the geo-referenced orthophotos before being horizontally rotated and plot-centered cropped into seven non-overlapping and square image clippings. Due to orthophoto corruptions and destructive field measurements, some sections were manually removed, resulting in 21,371 images for Mixed-CKA and 18 800 images for Mixed-WG. For Mixed-WG, a significant spatial alignment error was noticed by visual inspection, which is up to 10cm, but inconsistent across the images and, therefore, difficult to filter out. Since 10cm corresponds approximately to the spatial extent of a faba bean plant at 20 days after sowing (DAS), the offset is well visible in the early images. For this reason, Mixed-WG is not used for training; instead, it is intended to check the transferability, i.e., model learned on Mixed-CKA and applied on Mixed-WG.

In addition, a variety of other data were collected in this crop mixture experiment, including weather, soil, and nutrient parameters as well as height and biomass measurements [26, 27] that are used in this work to calibrate and evaluate a process-based crop growth model as described in the Process-based modeling of crop mixtures section. In the following experiments, the manual biomass cuts on day 83 after sowing are referred to as the “cutting reference”.

The datasets vary in complexity: The challenges of the GrowliFlower and MixedCrop datasets are the considerable gaps of different lengths between the recording times. In addition, there are large spectral variations both within each time series and between Mixed-CKA and Mixed-WG, mainly due to different solar radiations, cloud coverings, and soil moistures during the overflights. Compared to the other datasets, MixedCrop is the most challenging due to its small size combined with many overlapping mixed crops, even at early growth stages. All images are resized to a uniform size of ${256}~{\textrm{px}} \times {256}~{\textrm{px}}$ for the experiments, resulting in different ground sample distances (GSD) from 0.23 mm to 5.67 mm. The image sequences are divided into the same spatially separated training, validation, and test sets for all experiments.

### Image ﻿generation

For image generation, we build a multi-conditional Wasserstein GAN with gradient penalty (CWGAN-GP) [28] from several state-of-the-art components. The network consists of a generator $\mathcal {G}_\theta$ and a critic $\mathcal {D}_\delta$, where $\mathcal {G}_\theta$ predicts images and $\mathcal {D}_\delta$ estimates a score for generated and real images. A special focus is on integrating multiple conditions of different types in the architecture as described in the Network architecture with multi-conditioning section.

#### Conditional wasserstein GAN objective

In the generator, a target image ${{\textsf {\textit{X}}}}_{\text {gen}}=\mathcal {G}_\theta ({{\textsf {\textit{X}}}}_{\text {in}}, {\varvec{y}},{\varvec{z}})$ is generated from an input image ${{\textsf {\textit{X}}}}_{\text {in}}$, conditions ${\varvec{y}}$ that split into $[{\varvec{y}}_\text {in},{\varvec{y}}_\text {gen}]$, and noise ${\varvec{z}}\sim \mathcal {N}(0,1)$. Both ${\varvec{y}}_\text {in}$ and ${\varvec{y}}_\text {gen}$ represent multi-conditioning, which can be composed of several of the following conditions: categorical (class) variables *c*, discrete variables *t*, and continuous variables $\varvec{b}$. In the critic, either the reference $\mathcal {D}_\delta ({{\textsf {\textit{X}}}}_{\text {ref}},{{\textsf {\textit{X}}}}_{\text {in}},$ $\varvec{y}$) or the generated image $\mathcal {D}_\delta ({{\textsf {\textit{X}}}}_{\text {gen}},{{\textsf {\textit{X}}}}_{\text {in}},$ $\varvec{y}$) are presented along with input image and conditions. The critic estimates a score for both real and generated input, which is capable of enforcing the minimization of the Wasserstein distance between the two distributions. The objective of adversarial training is to optimize the parameters $\theta$ and $\delta$ by maximizing the objective function $L_{\text {GAN}}(\mathcal {G}_\theta ,\mathcal {D}_\delta )$ by $\mathcal {D}_\delta$ and minimizing it by $\mathcal {G}_\theta$.

1 $$\begin{aligned} \theta ^*, \delta ^* = \arg \min _\theta \arg \max _\delta L_{\text {GAN}}(\mathcal {G}_\theta ,\mathcal {D}_\delta ) \end{aligned}$$

(2) represents $L_{\text {GAN}}(\mathcal {G}_\theta ,\mathcal {D}_\delta )$ with the classic CWGAN objective in the first line [29], added with the gradient penalty term in the second line to enforce the required 1-Lipschitz continuity of $\mathcal {D}_\delta$ [28].

2 $$\begin{aligned} \begin{aligned} L_{\text {GAN}}(\mathcal {G}_\theta , \mathcal {D}_\delta ) = \mathbb {E}_{{({{\varvec{z}}},{{\textsf {\textit{X}}}}_{\text {in}}},{{{\varvec{y}}})}}&[\mathcal {D}_\delta (\mathcal {G}_\theta ({{\textsf {\textit{X}}}}_{\text {in}},{\varvec{y}},{\varvec{z}}),{{\textsf {\textit{X}}}}_{\text {in}},{\varvec{y}})] - \mathbb {E}_{({{\textsf {\textit{X}}}}_{\text {ref}},{{\textsf {\textit{X}}}}_{\text {in}},{{{\varvec{y}}}})} [\mathcal {D}_\delta ({{\textsf {\textit{X}}}}_{\text {ref}},{{\textsf {\textit{X}}}}_{\text {in}},{\varvec{y}})]\\&+ \lambda _{\text {GP}}\mathbb {E}_{({{\textsf {\textit{X}}}}_{\text {in}},\hat{{{\textsf {\textit{X}}}}})} [(\Vert \nabla _{\hat{{{\textsf {\textit{X}}}}}}\mathcal {D}_\delta ({{\textsf {\textit{X}}}}_{\text {in}},\hat{{{\textsf {\textit{X}}}}})\Vert _{2}-1)^2] \end{aligned} \end{aligned}$$

The gradient penalty is computed by blending a generated image with a reference image, resulting in $\hat{{{\textsf {\textit{X}}}}}=\epsilon {{\textsf {\textit{X}}}}_{\text {ref}}+(1-\epsilon )\mathcal {G}_\theta ({{\textsf {\textit{X}}}}_{\text {in}},{\varvec{y}},{\varvec{z}})$, where $\epsilon$ is a random value in the range [0, 1], and its impact is controlled by $\lambda _{\text {GP}}$. Using $L_{\text {GAN}}(\mathcal {G}_\theta , \mathcal {D}_\delta )$ minimizes the Wasserstein-1 distance, sidestepping issues like mode collapse and vanishing gradients in classic GAN training.

#### Network architecture with multi-conditioning

*Generator* The generator consists of an encoder $\mathcal {P}$ that compresses the input image and conditions related to the input image into a latent representation $\xi =\mathcal {P}({{\textsf {\textit{X}}}}_{\text {in}},{\varvec{y}}_{\text {in}})$ and a decoder $\mathcal {Q}$ that generates the target image from this latent representation, the conditions for the image to be generated and a stochastic component ${{\textsf {\textit{X}}}}_{\text {gen}} = \mathcal {Q}(\xi ,{\varvec{y}}_{\text {gen}},{\varvec{z}})$. While for image encoding a ResNet-18 backbone [30] without final fully connected layer and global average pooling is used, decoding works architecturally inverse to that. To integrate the conditions, all batch normalization layers are replaced by conditional batch normalization layers (CBN) [31], where the learnable affine parameters of classical batch normalization layers [32] are conditioned on some auxiliary variable $\varvec{a}$. In our case, $\varvec{a}$ are embeddings of the conditions $\varvec{y}$ using an embedding function $\Phi$. In particular, the encoder’s CBN layers are conditioned on the embeddings related to the input image ${\varvec{a}}_\text {in} = \Phi ({\varvec{y}}_\text {in})$. In contrast, the decoder’s CBN layers are conditioned on the embeddings related to the image to be generated ${\varvec{a}}_\text {gen} = \Phi ({\varvec{y}}_\text {gen})$. Specifically, the embedding function is condition-type-specific since $\varvec{y}$ can consist of conditions of up to 3 different types: discrete temporal information *t*, categorical class information *c*, and continuous variables ${\varvec{b}}$. So individual embeddings are performed for each type of condition in $\varvec{y}$, which are then concatenated to $\varvec{a}$.

3 $$\begin{aligned} &{\varvec{y}}_{\text {in}} = [t_{\text {in}},c_{\text {in}},{\varvec{b}}_{\text {in}}],\\ & {\varvec{y}}_{\text {gen}} = [t_{\text {gen}},c_{\text {gen}},{\varvec{b}}_{\text {gen}}] \\ &{\varvec{a}}_{\text {in}} = [\Phi _t(t_{\text {in}}),\Phi _c(c_{\text {in}}),\Phi _b({\varvec{b}}_{\text {in}})], \\ &  {\varvec{a}}_{\text {gen}} = [\Phi _t(t_{\text {gen}}),\Phi _c(c_{\text {gen}}),\Phi _b({\varvec{b}}_{\text{gen}})] \end{aligned}$$

Here, the temporal embedding $\Phi _t$ consists of positional encoding of discrete time points followed by a two-layer MLP with a sigmoid linear unit (SiLU) function in between. The class embedding $\Phi _c$ represents a classic lookup table embedding that maps indices of categorical class variables to a continuous vector representation. To embed a vector of continuous values in $\Phi _b$, a two-layer MLP with SiLU function in between is used. In the experiments, the conditions *c* and $\varvec{b}$ are not always used, then embedding and resp. concatenating of unused conditions is omitted. Notably, $t_{\text {in}}$/$t_{\text {gen}}$ and $c_{\text {in}}$/$c_{\text {gen}}$ are scalars representing in this work time (*t*) and treatment (*c*), respectively, while ${\varvec{b}}_{\text {in}}$/${\varvec{b}}_{\text {gen}}$ are vectors, and in this work are 2-dimensional due to SW and FB biomass. However, after embedding the individual components of $\varvec{y}$, it is ensured that $\Phi _t({\varvec{t}})$, $\Phi _c({\varvec{c}})$, and $\Phi _a({\varvec{b}})$ all represent continuous vectors of the same 64-dimensional embedding size, which avoid prior weighting of different conditions. Besides, CBN has already included a linear embedding for all conditions, but the additional condition-type-specific embedding has stabilized the training process.

To also incorporate stochasticity into the network, a random 128-dim noise vector ${\varvec{z}}\sim \mathcal {N}(0,1) \in \mathcal {Z}$ is generated and via noise mapping network  inspired by StyleGAN [33] projected to the latent code ${\varvec{w}} \in \mathcal {W}$, that matches the channel dimension of the latent representation $\xi$. The mapping network  is a shallow three-layer linear embedding network, which gradually projects the 128-dimensional $\varvec{z}$ to the 512-dimensional $\varvec{w}$, which corresponds to the channel size of the ResNet-18 latent representation. After repeating $\varvec{w}$ for the spatial dimension (global average pooling is omitted), it is finally added to $\xi$.

*Critic* The critic takes either the generated ${{\textsf {\textit{X}}}}_{\text {gen}}$ or reference image ${{\textsf {\textit{X}}}}_{\text {ref}}$ along with the input image ${{\textsf {\textit{X}}}}_{\text {in}}$, and the conditions ${\varvec{y}}$ as input. The images are concatenated channel-wise in the input and initially passed through a convolutional layer and LeakyReLU activation. This is followed by several convolutional blocks consisting of a convolutional layer, instance normalization, and LeakyReLU up to a spatial dimension of $[16\times 16]$. Since batch normalization should be avoided in the Wasserstein critic [28], the conditions are not integrated in this case with CBN. Instead, each condition is first embedded to dimension 256 with a different embedding function $\Psi$ than $\Phi$ in the generator, but the architecture of the embedding functions inside $\Psi$ and $\Phi$ are the same. Then, embedded conditions are reshaped, and channel-wise concatenated to the intermediate critic representation of spatial size $[16\times 16]$. Note that here, the conditions of both the input and the image to be generated are concatenated. From this concatenated representation, the final score is generated with further convolutional blocks. Previous experiments have shown that the training converges significantly better with an intermediate fusion of the conditions than with a fusion directly in the critic input.

#### Optimization and hyperparameter

The data sampling is special since multiple reference images can be used for every input image due to the possibility of temporal conditioning. Thus, in each epoch, we first iterate classically over all training images, which are then used as input images. Second, always another random image of the same plant is sampled for each input image, representing the reference plant. The conditions ${\varvec{y}}_{\text {in}}$ and ${\varvec{y}}_{\text {gen}}$ are drawn according to the sampled images. This causes that during the training, $c_{\text {in}}=c_{\text {gen}}$ because the treatment class does not change over time. To calculate test scores, the sampling procedure is identical, i.e., each test image represents an input image once and gets assigned a random growth stage as the reference image to be generated. For inference, the conditions can be varied arbitrarily, what we call data-driven simulation. So a treatment change $c_{\text {in}}\ne c_{\text {gen}}$ is possible, $\varvec{b}$ does not have to fit the reference values, and *t* can deviate from the training range.

Adam optimizer is used with a learning rate of 1e-4 for both $\mathcal {G}_\theta$ and $\mathcal {D}_\delta$ optimization. Regardless of the number of conditions, the models are trained for 5000 epochs, after which the best epoch is selected based on the lowest LPIPS on the validation data. As image augmentations, horizontal and vertical flipping, ${90}^{\circ }$ rotations, slight translations within a random affine transformation, and ShadowOut, which is a semi-transparent version of CutOut [34], are applied simultaneously to input and reference or generated image. Using a single NVIDIA A100-PCIE-40GB and a batch size of 64, the training duration is between 13 day and 35 day, depending on the dataset size.

#### Evaluation of image quality

To evaluate the quality of the generated images, we use a well-established set of GAN evaluation metrics. For the direct comparison between generated and reference images of the same time point, we use the Multi-scale Structural Similarity Index Measure (MS-SSIM [35], optimal: 1) and the Learned Perceptual Image Patch Similarity (LPIPS [36], optimal: 0). While MS-SSIM compares the generated with the reference image directly at different resolutions of the image space, LPIPS evaluates the similarity of image patch activations in the VGG-embedded latent space, which has been shown to have a high correlation with human perception. In addition, the Fréchet Inception Distance (FID [37], optimal: 0) is used to compare not only the quality but also the diversity of the generated image distribution with the real image distribution of the test dataset. However, for long-term predictions far into the future or past, generated and reference images are not expected to match at the pixel level. This is because of the significant difference in the growth stage of the input image and the image to be generated. Although FID will degrade less as long as the plants fit into the distribution of each growth stage, poor results are expected for MS-SSIM and LPIPS in such cases. To evaluate whether useful plant-related traits can still be derived, we use growth estimation models, which determine leaf area (see Estimation of projected leaf area section) and biomass (see Estimation of biomass section) from the generated images.

### Growth estimation

Depending on the dataset and plant type, growth estimation is realized by instance segmentation to estimate projected leaf area or by image regression to estimate biomass. Both can also be considered plant phenotyping based on state-of-the-art neural networks.

#### Estimation of projected leaf area

For Arabidopsis and GrowliFlower, growth is determined using the plant trait projected leaf area (PLA). Both datasets are well suited for this purpose because different plants do not overlap until advanced growth stages. The PLA is derived as an image-wise pixel sum of plant segmentations predicted with a Mask R-CNN instance segmentation model [38]. For this, two models, with pre-trained ImageNet weights [39], are fine-tuned on a few images of the respective plant dataset, for which reference segmentation masks are available. By multiplying the PLA with the squared dataset-dependent ground sample distance (GSD), we report PLA in the unit $\hbox {mm}^{2}$ for Arabidopsis and $\hbox {cm}^{2}$ for GrowliFlower or for comparability normalized in units of %/image, which is achieved by dividing the PLA by the image size. In this work, PLA is not calculated for the whole image but only out of the segmentation predictions for the center plant, which is especially relevant for GrowliFlower, where there are, in most cases, multiple plants per image. To compare the PLA of a single generated and reference image pair, we use $\Delta \text {PLA}=\text {PLA}^{\text {gen}}-\text {PLA}^{\text {ref}}$. For MixedCrop, PLA cannot be extracted with sufficient accuracy at the pixel level for the individual crop species due to the fine structure of the wheat ears, enormous plant overlap, and a lack of annotated images [40]. The accuracy evaluation of the trained instance segmentation models can be found in the Accuracy of projected leaf area estimation section.

#### Estimation of biomass

Instead of PLA, for MixedCrop, dried biomass (BM) in tons per hectare [$\hbox {t ha}^{-1}$] is to be derived from the images as a growth indicator, divided into the two mixture species spring wheat (SW) and faba bean (FB). To estimate both with one model, a ResNet-18 [30] is used, modifying the last linear layer to two output neurons, which are activated with ReLU since only positive biomass values are possible. The mean squared error (MSE) function is used as the loss function. We use weights from a pre-training with ImageNet [39] and fine-tune on MixedCrop images and corresponding reference biomass values. These reference biomass values are not actual in-field measurements but come from a process-based crop growth model for mixtures (see Process-based modeling of crop mixtures section) that provides simulated SW and FB biomasses for each image time point. Notably, we use the same simulated biomass values used as conditions in the image generation part of the framework. However, this dual use is methodologically not critical since the image generation and growth estimation parts are trained independently of each other. Similar to PLA, we use $\Delta \text {BM}=\text {BM}^{\text {gen}}-\text {BM}^{\text {ref}}$ to report biomass deviations between two images. Overall, estimating biomass from bird’s eye view imagery has three main challenges and inherent sources of error. First, biomass is a 3D quantity derived from 2D images. Second, the process-based crop growth model only estimates dried biomass (dry matter) for all growth stages, which is used as a reference for training the growth estimation model. However, the images show plants with their actual humidity (fresh matter), which changes over time. Third, the simulation result varies only treatment-wise, but even replicates of the same treatments have developed differently in the field due to different soil conditions and random effects. For the discussion about the biomass estimation results and accuracies, see Accuracy of biomass estimation section.

In the evaluation for a whole test set with *N* images, the mean absolute error (MAE) and the mean error (ME) are calculated as follows between plant traits (PT) of the generated and the reference image, whereby either PLA or BM serve as PT.

4 $$\begin{aligned} \text {MAE}=\frac{\sum _{i=1}^N |\text {PT}^{\text {gen}}_i-\text {PT}^{\text {ref}}_i|}{N} \quad \text {and}\quad \text {ME}=\frac{\sum _{i=1}^N \text {PT}^{\text {gen}}_i-\text {PT}^{\text {ref}}_i}{N} \end{aligned}$$

Here, the quantity measure ME indicates whether the PT is overall underestimated (ME negative) or overestimated (ME positive). For whole agricultural fields, the mean error (ME) is informative, in case it is not as important to accurately determine the yield of individual field regions but rather to evaluate whether the overall mean predictive error for the entire field is low.

#### Process-based modeling of crop mixtures

The process-based crop growth simulations were conducted in SIMPLACE (Scientific Impact Assessment and Modeling Platform for Advanced Crop Ecosystem Management) [41]. Different sub-models in the SIMPLACE framework, called “SimComponents”, were combined, namely LINTULPhenology, LINTUL5NPKDemand, SlimNitrogen, LINTUL5Biomass, SlimRoots, and SlimWater, amongst others. An overview of key SimComponents^2^ is described in Seidel et al. [42]. Specifically, the biomass per species was calculated by SimComponent LINTUL5Biomass, which considers water and nitrogen limitation effects on biomass increment. The mixture model was developed in the SIMPLACE framework and simulates the splitting of solar radiation according to the competition of the two species and the water and nitrogen uptake of two crop species planted in a mixture. The model was calibrated and tested on three environments (CKA 2020, 2021, and WG 2020) based on collected data from the crops cultivated solely and evaluated based on the data in the mixture treatments.

## Results and discussions

In this section, the results of the growth estimation models are described at the beginning, as the accuracies of these models are needed to discuss the image generation results. In the following, we first show the results of image generation with only temporal variation, which allows a comparison with reference data, then simulations with further changed conditions, and finally, the transferability to another experimental site.

### Accuracy of projected leaf area estimation

**Table 2:** Mask R-CNN instance segmentation accuracies for the real (non-generated) images of the test set divided into bounding box and segmentation. Overall average precision (AP), with thresholds at $\text {IoU}=0.50$ and $\text {IoU}=0.75$, and overall average recall (AR) are given

	Bounding box				Segmentation			
	AP	AP@0.50	AP@0.75	AR	AP	AP@0.50	AP@0.75	AR
Arabidopsis	0.92	0.99	0.99	0.95	0.77	0.99	0.98	0.78
GrowliFlower	0.86	0.96	0.92	0.88	0.78	0.97	0.92	0.82

Instance segmentation, used to derive PLA (projected leaf area), is trained on a small subset of the corresponding datasets for which reference segmentation masks are available. The exact numbers for all datasets are at the bottom of Table 1. The reference masks of the specified test set are used to run the evaluation in Table 2. It shows the instance segmentation accuracies using the measures AP and AR, which correlates with the PLA’s accuracy due to the direct derivation from these. The GrowliFlower accuracies are comparable to the results of Kierdorf et al. [25], i.e., sufficient to evaluate cauliflower growth. Arabidopsis has a higher AP and AR for bounding boxes and is at a comparable high level to GrowliFlower for segmentation; thus, it is also adequate to determine PLA.

### Accuracy of biomass estimation

**Table 3:** Biomass estimation accuracies assessed by MAE and ME between the estimations from the real (non-generated) images of the test set and the values from the process-based predictions

		SW/FB Mix		SW mono		FB mono		Overall	
		MAE	ME	MAE	ME	MAE	ME	MAE	ME
Mixed-CKA	SW	0.142	− 0.006	0.188	− 0.074	0.001	0.001	0.142	− 0.026
	FB	0.125	− 0.008	0.017	0.017	0.179	0.052	0.097	0.005
Mixed-WG	SW	0.126	− 0.023	0.150	− 0.050	0.018	0.018	0.122	− 0.027
	FB	0.105	− 0.026	0.003	0.003	0.185	-0.046	0.082	− 0.019

The scores are separated for mixtures and SW resp. FB monocultural fields. All units are given in $\hbox {t ha}^{-1}.$

**Fig. 3:**
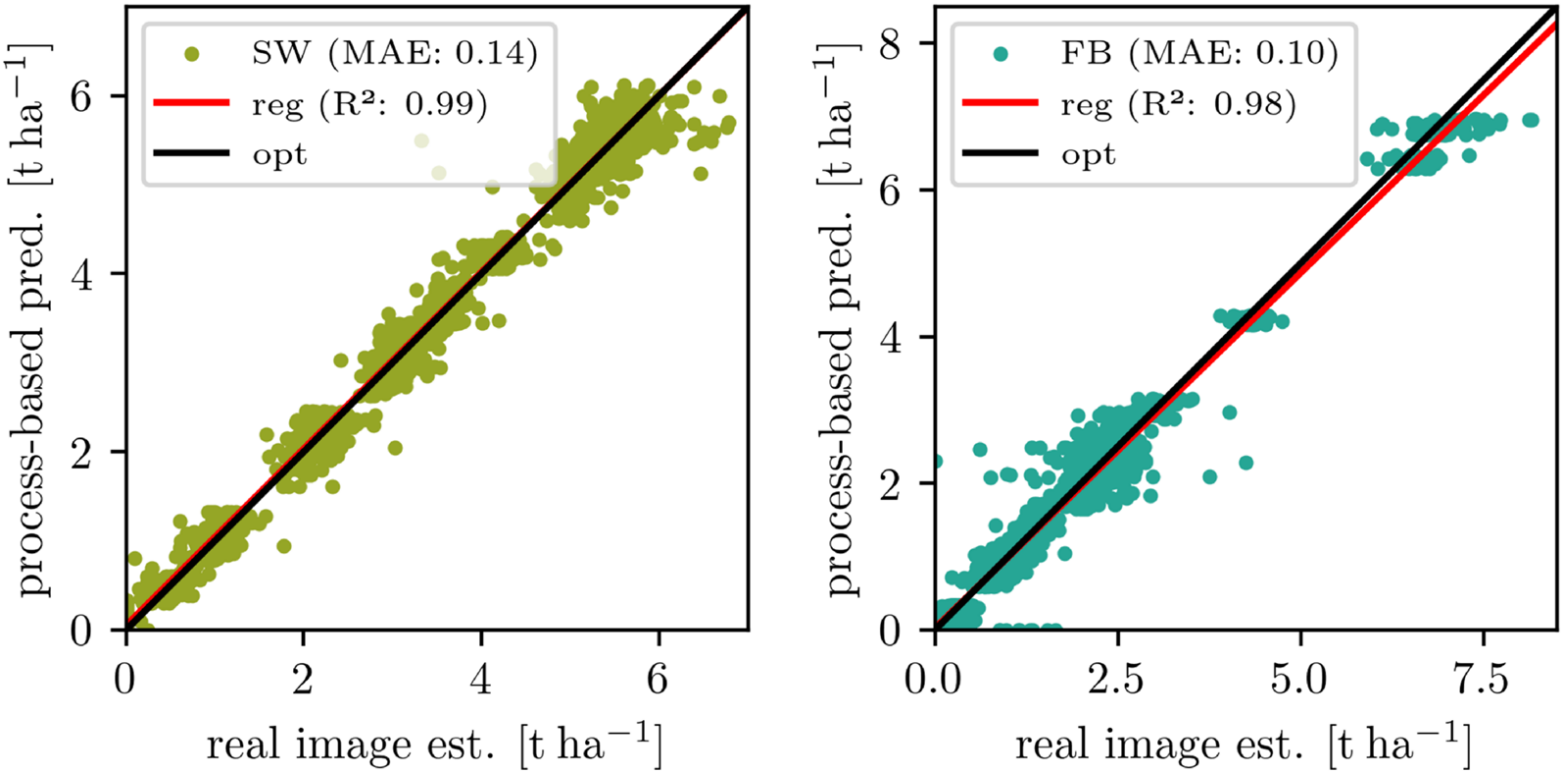
Scatter results of dried biomass estimation from real Mixed-CKA imagery overall growth stages and treatments (mixtures and monocultural fields) split up in spring wheat (SW) and faba bean (FB). The process-based predictions are used as a reference. The regression line is shown in red, and the optimal line is in black

The accuracy of dried biomass estimation for both MixedCrop sites is given in Table 3. For mixtures the MAE is between ${0.126}\,\hbox {t ha}^{-1}$ and $0.142\,\hbox {t ha}^{-1}$ for SW and between ${0.105}\,\hbox {t ha}^{-1}$ and $0.125\,\hbox {t ha}^{-1}$ for FB. Notably, the ME is less than $-0.01\,\hbox {t ha}^{-1}$ for mixtures at CKA and less than $-0.03\,\hbox {t ha}^{-1}$ at WG for both species. For the monoculture reference fields, the MAE is $0.179\,\hbox {t ha}^{-1}$ for FB in the FB monocultures and $0.188\,\hbox {t ha}^{-1}$ for SW in the SW monocultures. This is slightly higher than in the mixtures, which is expected because, in the monocultures, more of each species grows in absolute terms than in the mixtures. In return, the mixtures generally have a higher total biomass [26]. The low estimation of SW on FB monocultures between ${0.001}\,\hbox {t ha}^{-1}$ and ${0.018}\,\hbox {t ha}^{-1}$ and vice versa FB on SW monocultures between $0.003\,\hbox {t ha}^{-1}$ and $0.017\,\hbox {t ha}^{-1}$ can be considered as additional evidence that the model is able to distinguish the species with high accuracy. It can be assumed that a common weed found in both fields, *Chenopodium album*, which bears partial similarity to FB, is often incorrectly identified as FB. The mean absolute error (MAE) will be lower if there are fewer weeds or if it is included in the growth estimation model.

In Fig. 3, the overall results for CKA are visualized as two scatter plots for SW and FB, where the image-based estimations are plotted against the process-based predictions used as reference. The regression line is close to the optimal line with a minimal underestimation for SW ($\text {ME}={-0.026}\,\hbox {t ha}^{-1}$) and a minimal overestimation ($\text {ME}={0.005}\,\hbox {t ha}^{-1}$) for FB. In total, the regression results are $\text {MAE}={0.14}\,\hbox {t ha}^{-1}$ and ${ R }^{2}=0.99$ for SW and $\text {MAE}={0.10}\,\hbox {t ha}^{-1}$ and ${ R }^{2}=0.98$ for FB. With this, the model is considered accurate enough to evaluate generated images.

When assessing the following results, it is important to consider that they strongly rely on the accuracy of the growth estimation models. The accuracy of these models is evaluated solely based on real reference images. Any discrepancy between the growth estimation of real images and data-driven predictions  of the same growth stage can be attributed to two factors. Firstly, it could be due to actual differences in plant phenotypes compared to the reference images. This is the deviation we aim to identify. Secondly, part of the deviation may be caused by potential minor corruptions or artifacts in the artificial images, even if they pass GAN evaluation metrics. These corruptions can lead to incorrect estimations by the growth estimation model despite the visible plant phenotypes in the artificial images being accurate. This is because the growth estimation model was not trained on corrupted images. While it is hardly possible to avoid the second source of deviation completely, we strive to minimize it by augmenting the data used to train the growth estimation model, making it more robust and less susceptible to corruption. The magnitude of the deviation can be determined for certain growth stages by comparing the biomass estimation of real images with data-driven predictions of the same growth stage as shown on the right in Fig. 7.

### Time-varying image generation

**Table 4:** Evaluation with metrics MS-SSIM, LPIPS, and FID. Each row represents a distinct \ac{igm} trained on a varying combination of conditions time (t), treatment (trt), and simulated biomass (bm); for testing, only the input image and t are varied. MS-SSIM is reported for generations with different $|\Delta t|$ filters: $\text {T}_0$: identity $|\Delta t|=0$; ST: short-term $1\le |\Delta t|\le 10$; LT: long-term $|\Delta t|\ge 11.$

	Train conds.	MS-SSIM ($\uparrow$)				LPIPS ($\downarrow$)	FID ($\downarrow$)
	t trt bm	$\text {T}_0$	ST	LT	ø	ø	ø
Arabidopsis	$\checkmark$ $\times$ $\times$	0.94	0.81	0.68	0.80	0.25	6.54
GrowliFlower	$\checkmark$ $\times$ $\times$	0.98	0.30	0.20	0.29	0.51	20.17
Mixed-CKA	$\checkmark$ $\times$ $\times$	0.99	0.23	0.22	0.30	0.46	20.44
Mixed-CKA	$\checkmark$ $\checkmark$ $\times$	0.97	0.25	0.23	0.31	0.47	16.26
Mixed-CKA	$\checkmark$ $\checkmark$ $\checkmark$	0.99	0.23	0.22	0.29	0.46	24.86
Mixed-WG^1^	$\checkmark$ $\times$ $\times$	0.92	0.13	0.11	0.20	0.50	40.67

^1^ Transferability check: Model trained on Mixed-CKA and applied to Mixed-WG

**Table 5:** Plant-specific evaluation of projected leaf area (PLA) assessed by MAE and ME in the unit %/image. Both image generation models are trained solely on the temporal condition (t). MAE is reported for generations with different $|\Delta t|$ filters: $\text {T}_0$: identity $|\Delta t|=0$; ST: short-term $1\le |\Delta t|\le 10$; LT: long-term $|\Delta t|\ge 11$

	Train conds.	MAE				ME
	t trt bm	$\text {T}_0$	ST	LT	ø	ø
Arabidopsis	$\checkmark$ $\times$ $\times$	0.27	0.76	1.44	0.82	− 0.32
GrowliFlower	$\checkmark$ $\times$ $\times$	6.41	8.84	10.18	9.64	1.27

**Table 6:** Plant-specific evaluation of mixture biomasses (SW/FB) assessed by MAE and ME in the unit t ha^-1^ given for all (OA) and mixture (Mix) fields. Each row represents a distinct image generation model trained on a varying combination of conditions time (t), treatment (trt), and simulated biomass (bm); for testing, only the input image and t are varied. Overall fields, MAE is reported for generations with different $|\Delta t|$ filters: $\text {T}_0$: identity $|\Delta t|=0$; ST: short-term $1\le |\Delta t|\le 10$; LT: long-term $|\Delta t|\ge 11$

	Train		OA				OA	Mix	Mix
	conds.		MAE				ME	MAE	ME
	t trt bm		$\text {T}_0$	ST	LT	ø	ø	ø	ø
Mixed-CKA	$\checkmark$ $\times$ $\times$	SW	0.22	0.42	0.39	0.38	0.12	0.31	0.20
		FB	0.16	0.34	0.30	0.28	− 0.12	0.25	− 0.17
Mixed-CKA	$\checkmark$ $\checkmark$ $\times$	SW	0.30	0.22	0.25	0.24	0.09	0.25	0.15
		FB	0.24	0.16	0.19	0.19	− 0.13	0.24	− 0.15
Mixed-CKA	$\checkmark$ $\checkmark$ $\checkmark$	SW	0.17	0.21	0.18	0.18	− 0.02	0.18	0.05
		FB	0.11	0.16	0.14	0.13	− 0.01	0.15	− 0.04
Mixed-WG^1^	$\checkmark$ $\times$ $\times$	SW	0.45	1.25	1.14	1.07	0.18	1.06	0.24
		FB	0.41	0.48	0.67	0.64	− 0.04	0.62	− 0.11

^1^ Transferability check: Model trained on Mixed-CKA and applied to Mixed-WG

The first image generation experiment intends to evaluate how accurately our framework predicts images of other plant growth stages, given an input image and a different amount of conditions used for training, as indicated in Table 4. For each prediction, conditions that match the input image are used, and a varying prediction time and the corresponding reference image are randomly picked. Multiple models are trained on the different datasets and with a varying combination of conditions, namely time (t), treatment (trt), and simulated biomass (bm).

In Table 4, the predicted image quality is evaluated using the metrics MS-SSIM, LPIPS, and FID. Across all predictions, the highest accuracies are obtained with the Arabidopsis dataset for all three metrics $\text {MS-SSIM}=0.8$, $\text {LPIPS}=0.25$, and $\text {FID}=6.54$, while similarly lower overall accuracies are obtained with the GrowliFlower and MixedCrop datasets. For these, the MS-SSIM is between 0.29 and 0.31, LPIPS is between 0.46 and 0.51, and FID is between 16.26 and 24.86. Particularly remarkable is the dependence of the accuracy on the prediction distance, where MS-SSIM is higher for all datasets, the smaller $|\Delta t|$. In the case of $\Delta t = 0$, the model acts as an autoencoder, reproducing the input, also known as identity mapping. The identity mapping results show an MS-SSIM of 0.94 for Arabidopsis and MS-SSIM values between 0.97 and 0.99 for the Mixed-CKA models. From short-term (ST) to long-term (LT) predictions, the MS-SSIM continuously decreases to 0.20.

It is noticeable that Arabidopsis has better values in all metrics except $\text {T}_0$ than GrowliFlower and Mixed-CKA, which can be attributed to the daily recording times and controlled laboratory conditions with constant light and no weather effects. The identity mapping ($\text {T}_0$) is worse than the other datasets because, in the Arabidopsis dataset, multiple images were taken per day, which means it is not a strict identity mapping. However, this can be altered by changing the model time unit from days to hours. The MS-SSIM decrease from $\text {T}_0$ over ST to LT means the less far the model predicts into the future or past, the better the predicted images match the reference. Particularly, an MS-SSIM below 0.3 implies less similarity between predicted and reference images. In parallel, the FID for all models, including ST and LT predictions, is below 25, which can be considered good image quality. This is expected because, with increasing prediction steps, detailed plant phenotype appearances, like leaf counts and orientations, are increasingly difficult to predict. In contrast, general structural traits, like plant positions and overall sizes, can be predicted more accurately.

Insight into the usability of predicted images can be drawn from the plant-specific evaluation results using projected leaf area (PLA) estimation for Arabidopsis and GrowliFlower and biomass (BM) estimation for MixedCrop. Table 5 shows the obtained results for Arabidopsis and GrowliFlower in Table 5. It can be seen that MAE increases with larger $|\Delta t|$ in both cases, but the overall accuracy of ${<1}{\%}$ is high for Arabidopsis and with ${<10}{\%}$ slightly lower for GrowliFlower. In addition, for Arabidopsis, a mean error of ${-0.32}{\%}\approx {-11}\,\hbox {mm}^{2}$ indicates a small mean underestimation, while GrowliFlower heads are predicted larger $\text {ME}={1.27}{\%}\approx {80}\,\hbox {cm}^{2}$ than the corresponding reference.

The biomass evaluation for Mixed-CKA in Table 6 is divided into models trained with different conditions. All scores are given separately for SW and FB; moreover, an average over all plots and all mixture plots is reported. The MAE separation into different prediction distances shows that for $\text {T}_0$ the lowest deviations occur with a small increase to ST, but a decrease (accuracy gain) for LT over ST. The overall MAE ranges from ${0.13}\,\hbox {t ha}^{-1}$ to ${0.38}\,\hbox {t ha}^{-1}$ and is comparable to Mix MAE, where only mixtures are considered. Thereby, overall SW MAE is always higher than FB MAE with a magnitude of up to ${0.1}\,\hbox {t/h}$. Noticeably, overall FB ME is negative while SW ME is positive for all models except those trained on all conditions, showing a systematic SW over- and a FB underestimation. With an increasing number of conditions, the overall MAE decreases significantly by ${0.2}\,\hbox {t ha}^{-1}$ for SW and $0.15\,\hbox {t ha}^{-1}$ for FB. Comparing the accuracy when biomass estimation is performed on predicted mixtures (last two columns of Table 6) with the accuracy when it is performed on real mixtures (first two columns of Table 3) two results are shown: First, the MAE of the predicted mixtures using the model with all conditions is slightly above the MAE of the real mixtures (SW: $+{0.04}\,\hbox {t ha}^{-1}$, FB: $+0.03\,\hbox {t ha}^{-1}$). The other models trained with fewer conditions show higher deviations up to ${+0.17}\,\hbox {t ha}^{-1}$ for SW and ${+0.13}\,\hbox {t ha}^{-1}$ for FB. Second, the ME of the predicted mixtures using the model with all conditions is by a magnitude of 5 above the ME of the real mixtures.

We provide two assumptions for the SW and FB differences in MAE and ME: We assume that having for SW a generally higher MAE magnitude than for FB is caused by the higher absolute SW biomass level in the field. Additionally, we assume the reason for the systematic overestimation of SW and underestimation of FB (indicated by ME) is due to the unbalanced dataset: there are significantly more SW than FB monocultures. We assume that the image generation model copes worse with this unbalanced dataset than the growth estimation model, as FB plants are structurally more complex and therefore more readily quantifiable but more difficult to generate. Besides, MAE and ME decrease significantly as more conditions are added to the model. This can be explained by the model being better informed about the crop growth behavior if it receives more growth-influencing factors and can thus become more accurate. There is a loss of accuracy from identity mapping to short-term predictions but no significant loss from short-term to long-term predictions. Thus, long-term predictions can be considered valuable for phenotyping applications.

So, the quantitative evaluation leads to the overall finding: Although the predicted images match the reference images less at large $|\Delta t|$, they represent realistic plants of their respective growth stage, as indicated by FID, and are still accurate enough to derive reasonable plant traits, as indicated by plant-specific evaluation.

**Fig. 4:**
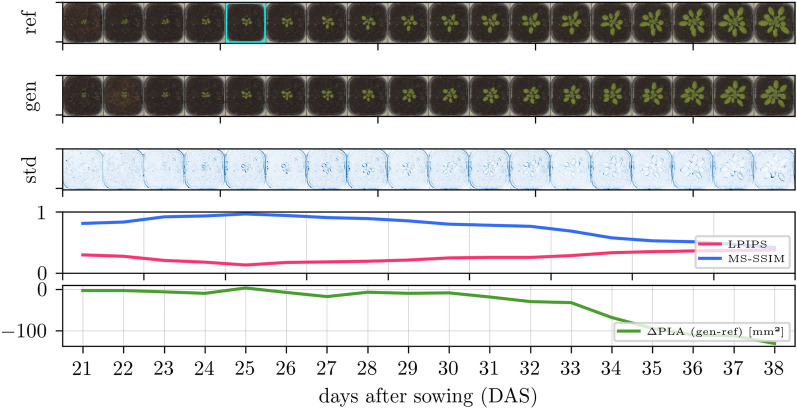
Time-varying image generation for Arabidopsis with, in the top row, reference images with an early growth stage as input (cyan frame), in the second row, all day-wise generated predictions, and, in the third row, standard deviation images over ten predictions with different noise input $\varvec{z}$ and otherwise constant input conditions. The two bottom rows have the quality metrics: learned perceptual image patch similarity (LPIPS), multiscale structural similarity (MS-SSIM), and the projected leaf area difference ($\Delta \hbox {PLA}$)

**Fig. 5:**
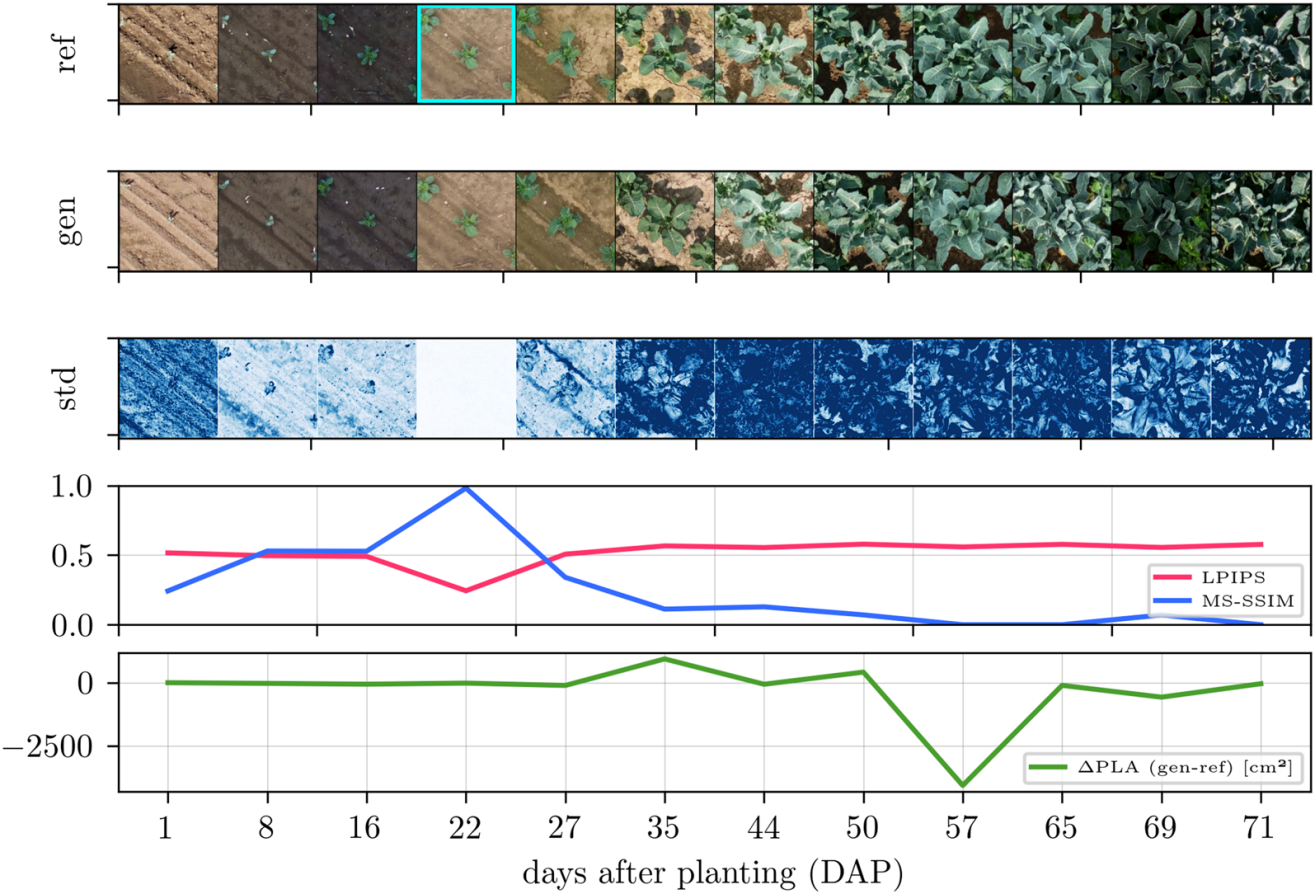
Time-varying image generation for GrowliFlower with, in the top row, reference images with an early growth stage as input (cyan frame), in the second row, all day-wise generated predictions, and, in the third row, standard deviation images over ten predictions with different noise input $\varvec{z}$ and otherwise constant input conditions. The two bottom rows show the quality metrics: learned perceptual image patch similarity (LPIPS), multiscale structural similarity (MS-SSIM), and the projected leaf area difference ($\Delta \hbox {PLA}$)

**Fig. 6:**
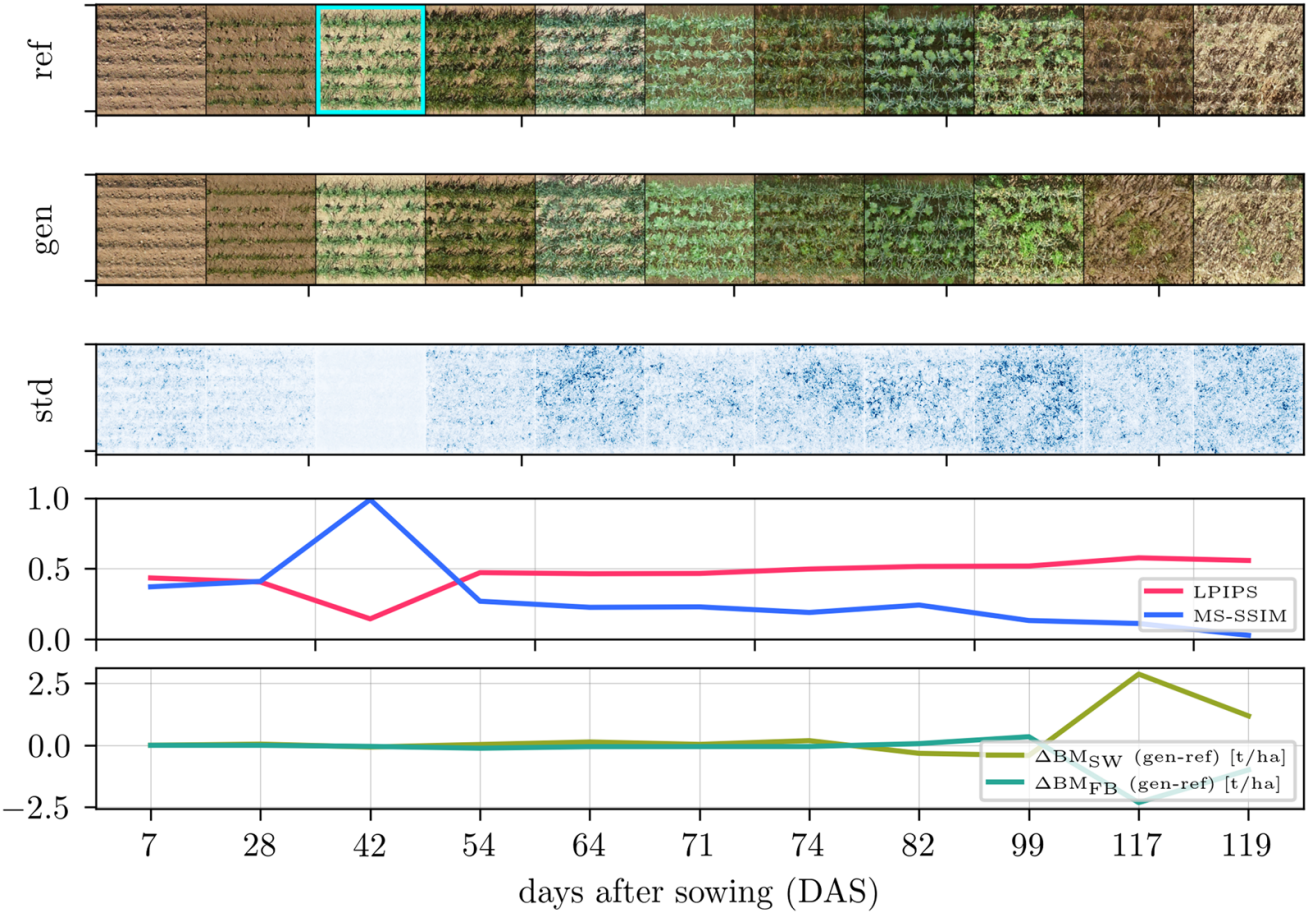
Time-varying image generation for Mixed-CKA with, in the top row, reference images with an early growth stage as input (cyan frame), in the second row, all day-wise generated predictions, and, in the third row, standard deviation images over ten predictions with different noise input $\varvec{z}$ and otherwise constant input conditions. The two bottom rows show the quality metrics: learned perceptual image patch similarity (LPIPS), multiscale structural similarity (MS-SSIM), and the biomass differences for spring wheat ($\Delta \text {BM}_\text {SW}$) and faba bean ($\Delta \text {BM}_\text {FB}$)

Further findings can be drawn from qualitative results showing selected time-varying image generation results in Fig. 4 for Arabidopsis, Fig. 5 for GrowliFlower, and Fig. 6 for Mixed-CKA, where models are used that are trained on the temporal condition only. Each figure consists of 5 rows: The first row contains a reference plant over time, where an early growth stage with a cyan frame is the input to the model in each case. The second row shows generated images by keeping except time all other conditions, including noise $\varvec{z}$, constant. The third row shows the variability image, which is the standard deviation over ten predictions of the same time point with different $\varvec{z}$, whereby the standard deviation is averaged over all RGB channels and overdrawn by a factor of four for clearer visualization. The darker the blue, the greater the variability for each pixel within the ten predictions. The fourth and fifth rows show each gen-ref image pair’s classical and plant-specific evaluation metrics.

For all datasets and time points, the predictions are realistic, with a few exceptions, such as the last image of GrowliFlower. Comparing the variability images, Arabidopsis has the lowest pixel-wise standard deviation, followed by MixedCrop and GrowliFlower. In all cases, there is high variability at the leaf edges, where the actual uncertainty is greatest. The LPIPS and MS-SSIM deteriorate with increasing $\Delta t$ with a peak each for identity mapping. Plant property curves differ for each data set: In Arabidopsis, $\Delta$PLA is close to zero until 30 DAS and then drifts into the negative range, indicating a leaf area underestimation for advanced growth stages. In GrowliFlower, the curve is close to zero with small fluctuations except for a large negative peak at 57 DAP, indicating that the leaf area could not be correctly estimated from the predicted image of this day. Similarly, for Mixed-CKA, the curves stay around zero until day 99, after which SW biomass is significantly overestimated with up to ${+2.5}\hbox {t ha}^{-1}$ and FB biomass is significantly underestimated with up to $-2.5\,\hbox {t ha}^{-1}$.

Two important insights emerge from the visualized images. First, a strong consistency of the generated images over time is given, which is visible in Arabidopsis and GrowliFlower through leaf orientations but also through neighboring plants and in Mixed-CKA through certain crop patterns such as small gaps (second crop row, right) or weeds (third and fourth crop row, center). Second, the dependence of the generated images on the input is visible for all datasets, particularly in the position of the plants and crop rows and by granules on the ground, which can be found on the input image as well as on several generated images. While the variability images show realistic uncertainties at the leaf edges, they also reveal a limitation in the image generation: While the identity mapping has no or extremely low variability, as expected, no continuous increase in variability over time is evident, leading to overconfidence at large $\Delta t$ where variability would be expected to be significantly higher. The parallel examination of MS-SSIM and LPIPS with the images confirms the findings from the quantitative results: Despite the images being less consistent with the reference as the prediction distance increases, there is neither a general visual quality decrease nor a general decrease in the accuracy of the estimated plant traits for time-varying predictions.

An overview of predictions for days not present in the datasets, so temporally out-of-distribution (OOD) can be found in Appendix B. While challenging due to large spectral differences between images of existing time points, it can be shown that realistic images can still be generated at new time points.

### Comparison of the process-based and the data-driven crop growth model

**Fig. 7:**
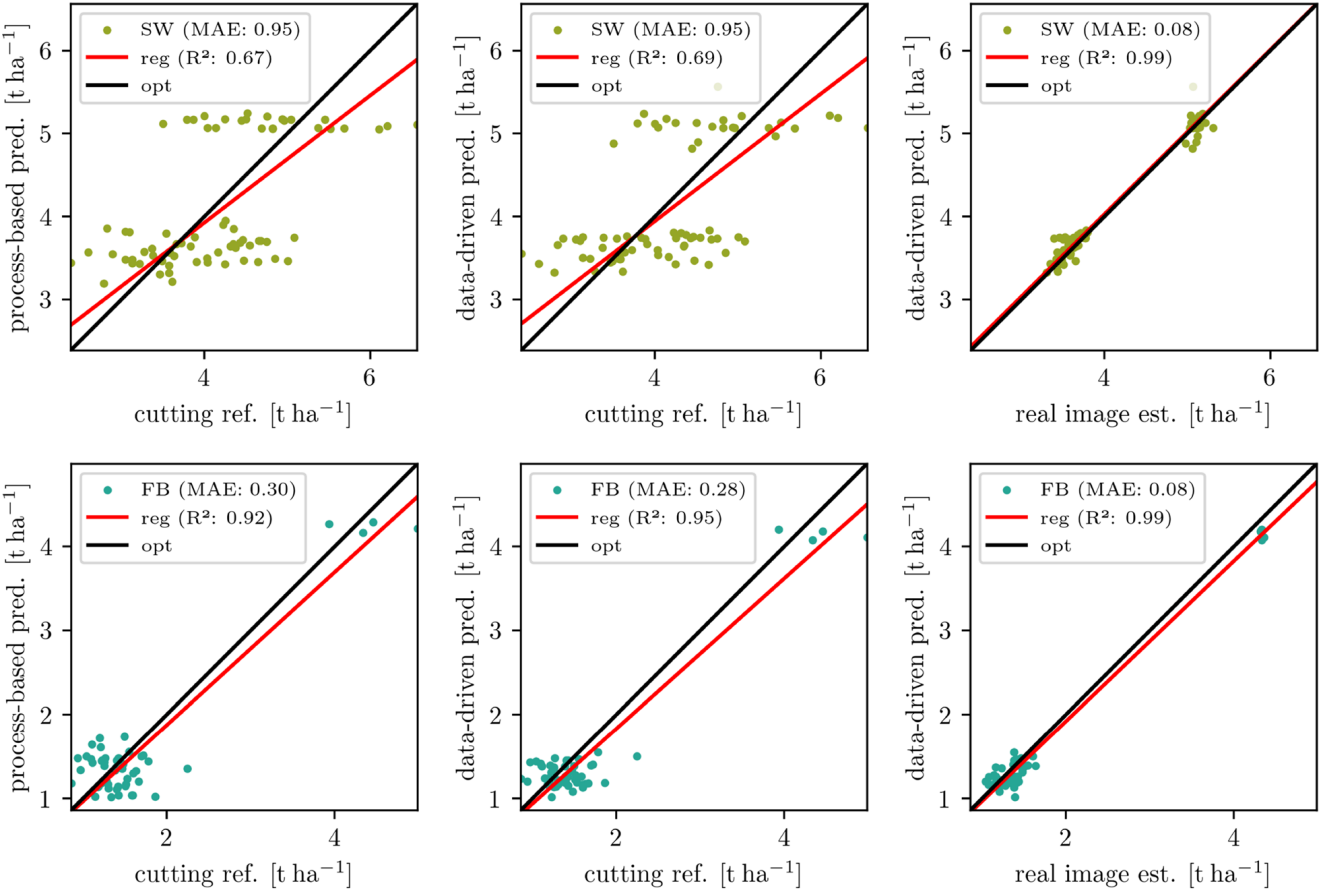
Scattering of model predictions for time ${82}\,\hbox {DAS}$ (left: process-based, middle: data-driven) with in-field biomass measurements (“cutting reference”) at time ${83}\,\hbox {DAS}$. On the right, the same data-driven predictions are compared with the estimates from real images at time ${82}\,\hbox {DAS}$

Since there are independent reference measurements of the dried biomass (“cutting reference”) for all plots at time ${83}\,\hbox {DAS}$ for Mixed-CKA, we can compare the process-based and the data-driven crop growth model predictions. For both models, we use the time point ${82}\,\hbox {DAS}$ after sowing as the prediction target, the closest image acquisition time before the biomass cuts. We select time point ${28}\,\hbox {DAS}$ as the image input of the data-driven model because it is the first time crops are recognizable on the images (cf. Fig. 2). As a further input condition, we use the treatment information, which is also available to the process-based model, but not the biomass information, which is only available retrospectively. Two aspects have to be taken into account in the comparison. Firstly, the starting conditions are not identical because the image-based model requires an input image from a previous growth stage. In contrast, the process-based model does not require an input image. Secondly, the models are not independent because the growth estimation part of the data-driven model was trained with the output of the process-based model. As a result, the data-driven model is expected to achieve at best the same accuracy as the process-based model when compared with the cutting reference, provided the generated images are of adequate quality. The latter is verified by comparing the estimated biomass from the data-driven prediction (generated images) with the estimated biomass from the real images from the reference day (82 DAS). If the data-driven model provides realistic predictions and the generated images are of a quality that is suitable for plant phenotyping, a high correlation can be expected.

In Fig. 7, the treatment-wise comparison between the process-based predictions and the cutting reference is shown on the left, between the data-driven predictions and the cutting reference in the middle, and between the data-driven predictions and the real image estimations on the right. The top row shows the SW and the bottom row the FB biomasses. Two clusters can be seen in all plots: The blob with the higher biomass contains the monocultures, while the lower biomass clusters represent the mixtures. The process-based model deviates from the cutting reference for SW with $\text {MAE}={0.95}\,\hbox {t ha}^{-1}$ (${ R }^{2}=0.67$) and for FB with $\text {MAE}={0.30}\,\hbox {t ha}^{-1}$ (${ R }^{2}=0.92$). The pattern is similar for the data-driven model, for SW with $\text {MAE}={0.95}\,\hbox {t ha}^{-1}$ (${ R }^{2}=0.69$) and for FB with $\text {MAE}={0.28}\,\hbox {t ha}^{-1}$ (${ R }^{2}=0.95$). Overall, there are significantly larger MAE for SW than for FB. In addition, the prediction range for SW is significantly narrower than the cutting reference range for both the process-based and the data-driven prediction. Focusing on the mixtures, the predicted values range between 3.2 and ${4}\,\hbox {t ha}^{-1}$ while for the cutting reference, they range between 2.4 and ${5}\,\hbox {t ha}^{-1}$. This means that the actual measured variability of SW biomass between treatments is significantly larger than the predicted variability, both process-based and data-driven. Remarkably, the mean value ${3.7}\,\hbox {t ha}^{-1}$ is identical for both models and the cutting reference. The comparison between the data-driven prediction and the estimation from the real images at time 82 DAS on the right side in Fig. 7 shows only a small $\text {MAE}={0.08}\,\hbox {t ha}^{-1}$ and a high ${ R }^{2}=0.99$ for both SW and FB. This means that the MAE for this time point is ${0.06}\,\hbox {t ha}^{-1}$ (SW) resp. ${0.02}\,\hbox {t ha}^{-1}$ (FB) lower than in the comparison of all time points of the process-based model with the estimation from real images (cf. Fig. 3). The red regression line indicates that overall SW is slightly over- and FB slightly underestimated, which is already analyzed in Time-varying image generation section.

Some findings can be taken away from the comparisons: Mainly: process-based and data-driven models achieve similar accuracy, despite the long-term prediction 54 days into the future. Both models can quantify differences between mixtures and monocultures of the same growth stage but are hardly sensitive to differences between the mixture treatments. They only achieve the prediction of a correct mean value, which can be explained by the fact that many cultivar differences occur randomly and are not significant [43]. In general, a machine learning model (growth estimation from images) can hardly be better than the training data (process-based output), which accounts for the similar pattern in the left and middle scatter plots. If there were other biomass reference data for each time point, we could use it to train the growth estimation model and become completely independent of the process-based model. It is conceivable that such biomass reference data might be available in the future and outperform the process-based model as it is trained with measurements instead of simulations. However, these biomass reference data would need to be available in advance and ideally be highly diverse to allow generalization across different environments.

### Data-driven simulation using treatment information

**Fig. 8:**
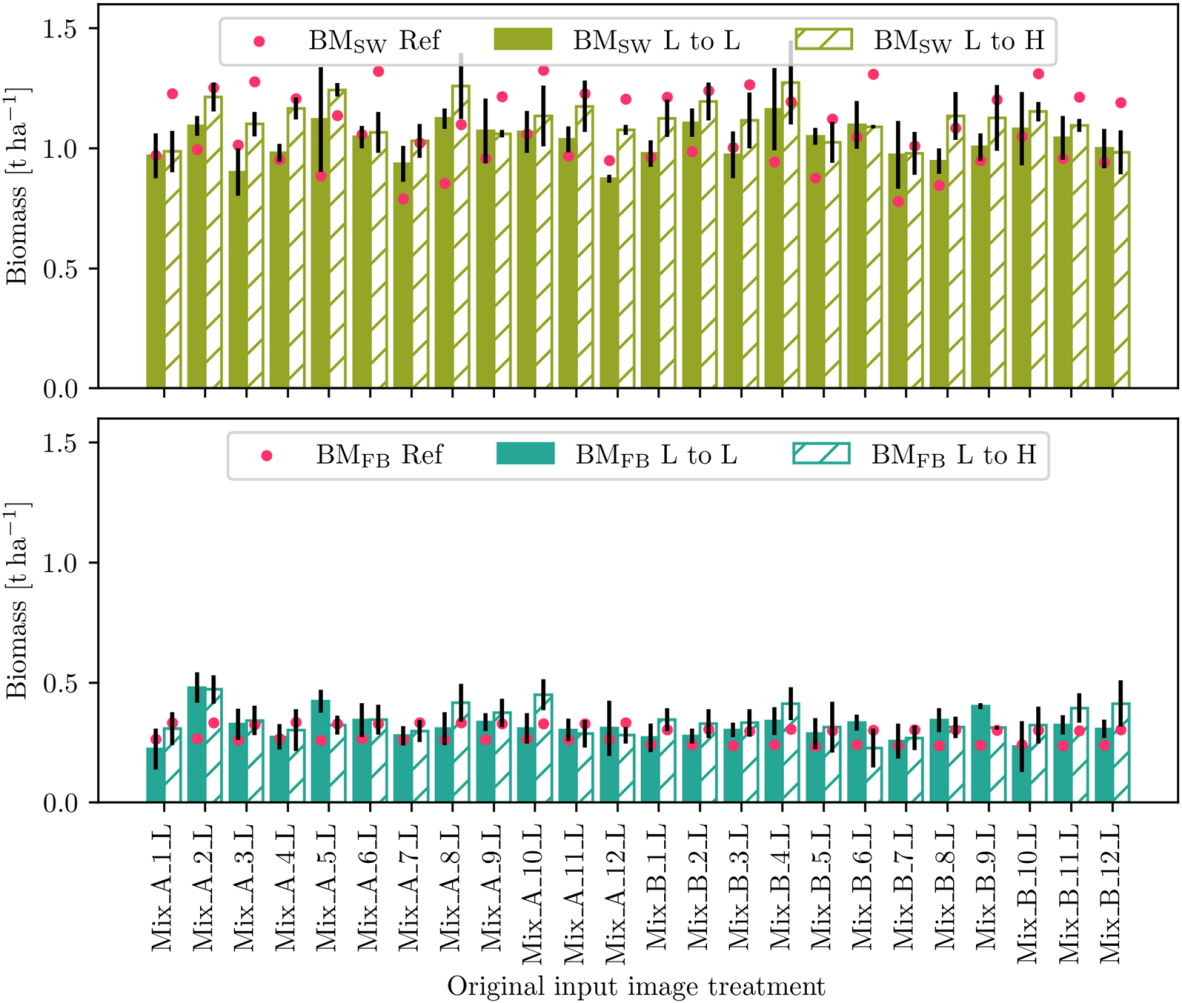
Simulating the SW (top) and FB (bottom) change from a low (L) density to a high (H) density treatment for all mixture field plots and the growth prediction step ${28}\,\hbox {DAS}$ to ${54}\,\hbox {DAS}$. While filled bars represent the comparative prediction under the original treatment, hashed bars represent the simulated treatment change. Black lines symbolize the standard deviation across treatment replicates; red dots symbolize the outcome of the process-based crop growth model for the resp. treatments and ${54}\,\hbox {DAS}$

**Fig. 9:**
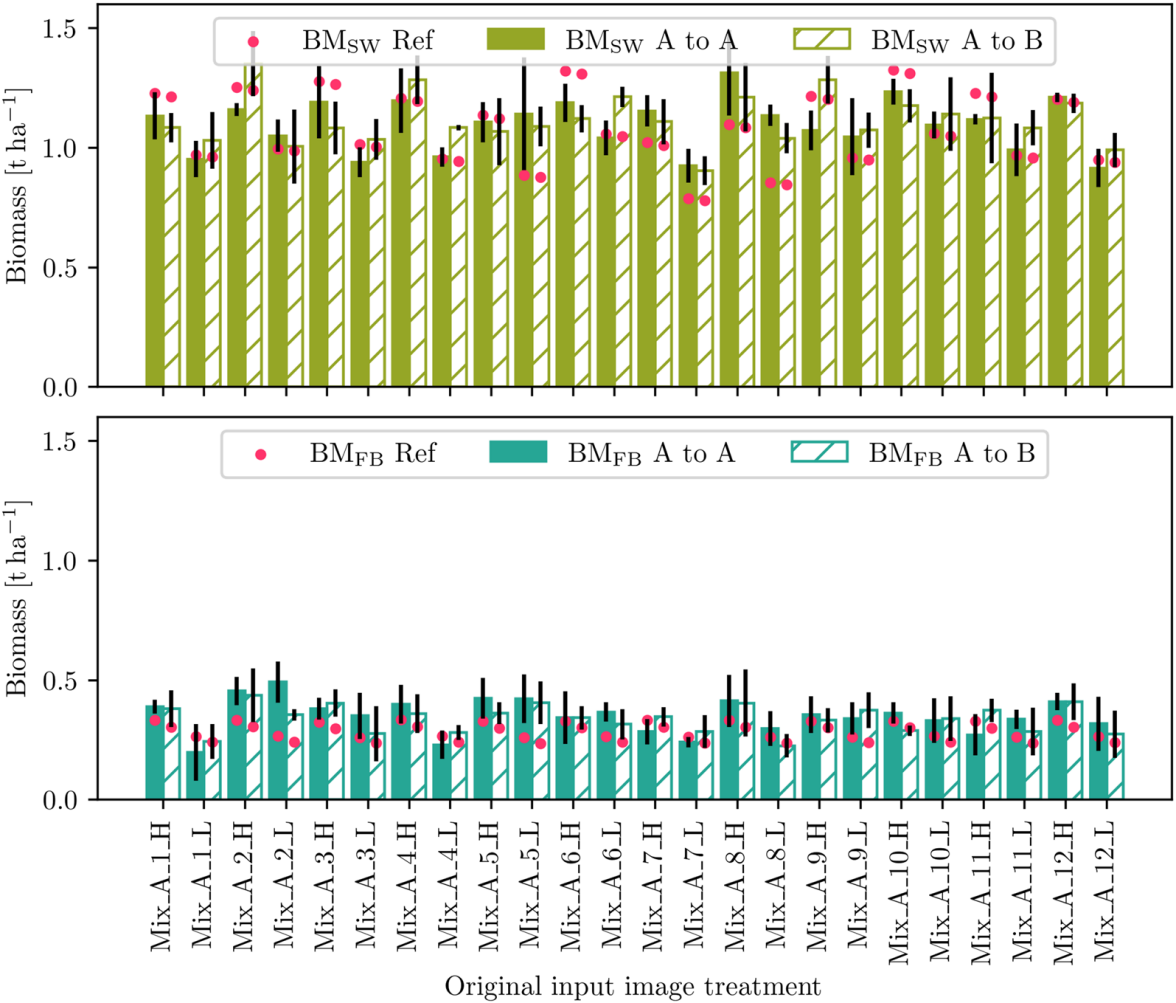
Simulating the SW (top) and FB (bottom) change from faba bean cultivar *Mallory* (A) to cultivar *Fanfare* (B) for all mixture field plots and the growth prediction step ${28}\,\hbox {DAS}$ to ${54}\,\hbox {DAS}$. While filled bars represent the comparative prediction under the original treatment, hashed bars represent the simulated treatment change. Black lines symbolize the standard deviation across treatment replicates; red dots symbolize the outcome of the process-based crop growth model for the resp. treatments and ${54}\,\hbox {DAS}$

The data-driven simulations on the MixedCrop dataset are intended to show the flexibility of the image generation model in the presence of changing growth-influencing variables. To enable an illustrative and informative demonstration and visualization, we systematically vary the time (t) and treatment (trt) information as a condition for the Mixed-CKA dataset. We use the results to investigate and evaluate how different treatments appear in the future when something about the treatment changes starting from a certain initial condition (image). We would like to emphasize that the change in treatments performed is intended to evaluate the method and is thus limited in its realistic nature, yet aims to show that our framework applies to realistic scenarios. We expect that the estimated biomass from the data-driven simulation changes in the same direction as that of the process-based plant growth model, confirming the reliability of the image generations.

In particular, two simulations are conducted from the input time point of ${28}\,\hbox {DAS}$ to ${54}\,\hbox {DAS}$ where first, the seed density is changed from low (L) to high (H) (Fig. 8), and second, the faba bean cultivar is changed from Mallory (A) to Fanfare (B) (Fig. 9). Thus, the input image is encoded in the original treatment, but a treatment change is made to decode the simulated future plant phenotype. The figures compare the data-driven prediction without treatment change (filled bars) with the prediction including treatment change (hashed bars) and the process-based predictions for the respective target treatment (red dots). The bars represent the treatment-wise mean, and the black lines are the standard deviation. We deliberately chose an early stage as the input because the differences in biomass between the treatments are not yet too great, and differences between the FB varieties are hardly discernible. However, we do not use DAS=7, which is bare soil, because we want to observe the spatial development of the crops. In addition, we focus on mixtures in the simulations to analyze the biomass of spring wheat and faba bean in parallel.

Focusing on the simulation of L $\rightarrow$ H in Fig. 8, the data-driven estimated biomass of the high-density simulated treatments (hashed bars) is higher than that of the low-density simulated ones (filled bars) for SW in 20/24 cases and for FB in 16/24 cases. The process-based biomass gain from L $\rightarrow$ H, shown by the red dots, is for SW significantly higher (${0.25}\,\hbox {t ha}^{-1}$) than for FB ($<0.1\,\hbox {t ha}^{-1}$). Averaged across all treatments, the biomass increases for both SW and FB. Apparently, FB biomass is slightly overestimated compared to the reference in almost all cases, and SW biomass is often overestimated for the L $\rightarrow$ L simulation while underestimated for L $\rightarrow$ H.

The analysis of the simulation of faba bean cultivar A $\rightarrow$ B in Fig. 9 is more challenging because only a small loss of biomass is expected for FB and an even smaller one for SW (almost the same level), as shown by the red dots. Treatment-wise, this decrease is not visible for either SW or FB: Only slightly more than half of the treatments is the hashed bar smaller than the filled bar for both SW (13/24) and FB (15/24). In average over all treatments, the hashed bars are smaller than the filled bares, albeit in the range of the standard deviation. Comparing high and low-density treatments, it can be seen that the estimated biomass from the high-density treatments is higher for SW in 10/12 cases and for FB in 7/12 cases.

Both simulation results show that even small changes in the growth-influencing factors affect the predicted images. Thereby, the reliability of the simulations is supported by the overall biomass increase from L $\rightarrow$ H treatments and decrease from faba bean cultivar A $\rightarrow$ B. If the biomass change (filled to hashed bar) for individual treatments does not correspond to the expected change (red dots), there are three possible interpretations. First, although the treatment condition is considered in the image generation model, its influence might not be strong enough, so the differences in the generated images are not sufficiently prominent. Second, the density resp. cultivar appearance of the input image might already be too prominent, making it difficult to change the growth stage later; e.g., plants cannot arise from anywhere. Third, the differences between low and high-density treatments resp. faba bean cultivars A and B are less clear in reality than the dynamic crop growth model suggests. In fact, the FB biomass gain for L $\rightarrow$ H and the FB/SW biomass loss for A $\rightarrow$ B is below the accuracy level of the biomass estimation (compare Table 6, which can explain why a clear trend in biomass changes is not particularly apparent for these cases. Apart from this specific experiment, we see the potential to simulate further treatment changes or their effects, e.g., weed cover. This varies over the growing season and can be estimated quickly in categorical measures (low, medium, high), allowing crop growth predictions adapted to current field conditions.

**Fig. 10:**
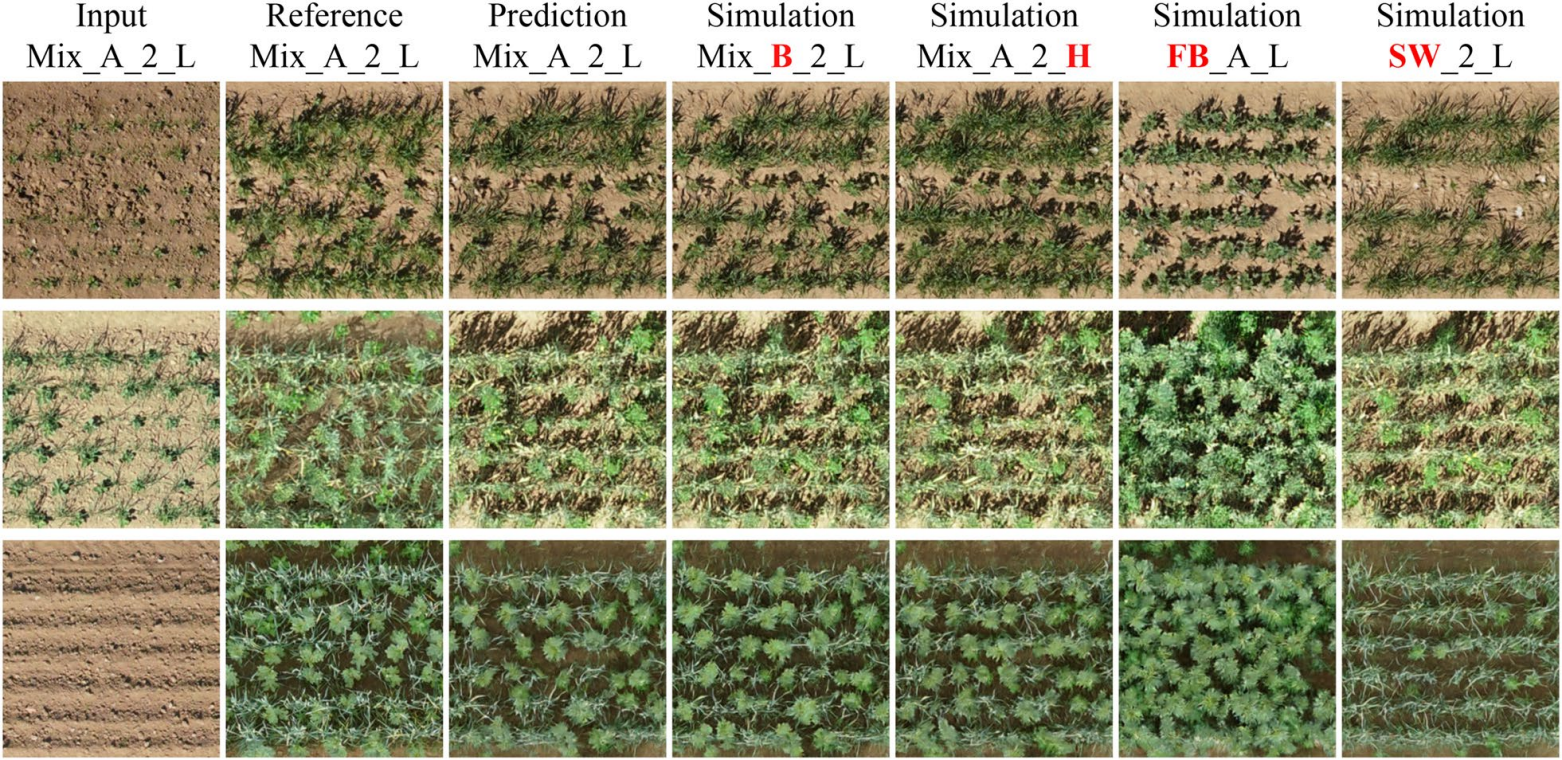
Growth simulation for different prediction steps and treatment changes in Mixed-CKA, first row ${28}\,\hbox {DAS}$ to ${45}\,\hbox {DAS}$, second row ${42}\,\hbox {DAS}$ to ${99}\,\hbox {DAS}$, and third row ${7}\,\hbox {DAS}$ to $82\,\hbox {DAS}$. The first column shows the input image, the second the corresponding reference image of the future growth stage, the third the predicted image at these treatment conditions, and columns 4 to 7 show simulations of change in faba bean cultivar, density, and to monocultural reference

Figure 10 also qualitatively illustrates the structural differences in the crop rows when simulating different treatments. Besides the growth prediction step from ${28}\,\hbox {DAS}$ to ${54}\,\hbox {DAS}$, two more growth prediction steps and two more treatment variations are simulated, including more unlikely scenarios, such as transformations of mixtures to monocultures. While such simulations rarely make sense from an application point of view, as long as a mixture component is not completely suppressed, it is nevertheless noteworthy to see the model visualizing such a treatment change if necessary.

### Data-driven simulation using process-based biomass

The following biomass simulation is intended to demonstrate the capability of including dynamic output variables of a process-based crop growth model in our framework. For this, we use the trained Mixed-CKA model on time (t), treatment (trt), and process-based simulated biomass (bm), whereby the biomass systematically varied to get predictions for different possible SW and FB biomass ratios. The time is randomly varied, so the simulation is performed over all growth stages by choosing a random prediction time point for each input mixture image and re-adjusting its biomass ratio. The starting point for the simulation is the biomasses calculated dynamically from the process-based crop growth model for each time point and treatment, $\text {BM}_\text {SW} = \text {BM}_\text {FB} = 100~\%$. While the image generation model was trained with a fixed biomass value attached to each reference image, we will demonstrate that almost any combination of biomass ratios can be chosen for inference as long as they are within the range of the training data.

**Fig. 11:**
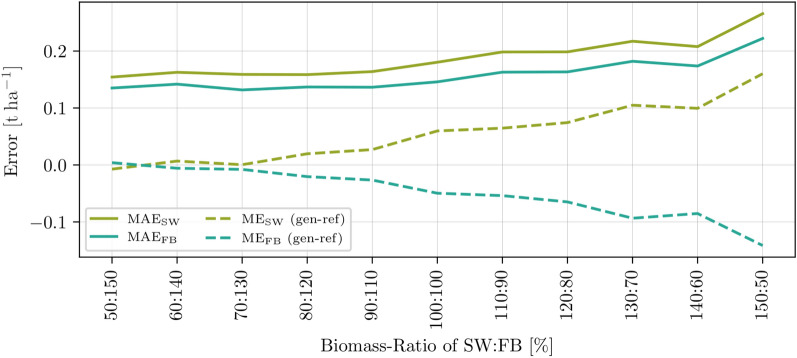
Comparing MAE and ME for image generations from $28\,\hbox {DAS}$ to ${54}\,\hbox {DAS}$ with different expected spring wheat (SW) to faba bean (FB) biomass ratios

Figure 11 shows MAE and ME respectively for SW and FB and different simulated biomass ratios, where the original composition (100:100) is shown in the middle, to the left, $\text {BM}_\text {FB}$ increases and to the right $\text {BM}_\text {SW}$. This is accordingly also noticeable in the ME: If the BM fraction for SW and FB increases, more biomass is also estimated in the predicted image and the ME increases. So $\text {ME}_\text {SW}$ rises to the right, and the $\text {ME}_\text {FB}$ rises to the left. The MAE reaches the minimum error where ME is also minimum at about the ratio 55 % SW to 145 % FB.

That a higher SW simulated biomass in the input of the framework also leads to a higher SW prediction in the output, for FB, accordingly, shows the reliability of our framework to generate predictions that realistically depend on the input conditions. It demonstrates the capability of our framework to generate images that plausibly explain the output of a process-based model. The minimum MAE/ME is not reached at 100:100, mainly due to the slight dataset bias towards SW and the resulting under-prediction of FB plants in the images, as already discussed. Assuming an unbiased image generation model, this type of analysis can serve to improve the calibration of the process-based model and bring it closer to image-based field observations: If the minimum MAE deviates from the expected minimum (in this case, 100:100), the process-based crop growth model could be adjusted in this direction or, in other words, complemented by the knowledge gained from the data-driven model. Note that other dynamic growth-influencing variables, like climatic conditions, can be used instead of process-based time-varying biomass, which could lead to even more feasible simulations.

### Transferability to new site

With a transferability experiment on the MixedCrop experiment, we aim to investigate the accuracy drop with which the model trained for Mixed-CKA, which takes time (t) as input condition, can be applied to the Mixed-WG site. The basic requirements are given by the same image size, resolution, crop species, and treatments (see Data section). However, this attempt to transfer the growth behavior of Mixed-CKA to images of Mixed-WG poses three main challenges. First, the growth behavior of conventionally managed CKA differs substantially from that of organically managed WG, as indicated, for instance, by weed abundance. Second, the spectral image properties are completely different for each time point, so both sites have their own “style”. Third, images were not taken simultaneously during the growing season at both locations, resulting in images from Mixed-WG being spatially and temporally out-of-distribution (OOD).

Tables 4 and 6 show the transferability quality measured by all evaluation metrics in the bottom line each. It can be seen that the results show significantly lower accuracies than the ones produced by models trained and tested on Mixed-CKA. However, the identity predictions still show a high MS-SSIM of 0.92.

The reason for the less accurate results lies in the first two aforementioned challenges, which lead to the predicted images not being well comparable to the reference images on a quantitative basis. Since the model only knows the style of CKA, but the reference images are in the style of WG, better scores were not expected. Focusing more on qualitative results, the third challenge of temporal OOD leads to corrupted results when the input image is significantly different from the style of the temporally nearest CKA image but is otherwise reliable, demonstrated in Appendix C. It shows both failed predictions and reasonable transfer examples, first for time points for which reference images are available, even if they do not match the reference, and second for the entire growing period.

For future experiments, the style could be added as an additional condition in the image generation model, or more generally with domain knowledge in the form of site-dependent context variables that influence style and plant growth itself [44]. While this requires a larger training dataset spanning multiple sites and styles, it will ensure even better transferability and help to merge multiple plant time series affected by various factors influencing factors into a more generic data-driven crop growth model.

## Conclusion

In this work, we have shown the capabilities of multi-conditional growth simulation using three datasets: Arabidopsis, GrowliFlower, and MixedCrop. For this purpose, in the first step, we combined several conditions of different types (discrete, continuous, categorical) in an image generation model, which is a conditional Wasserstein generative adversarial network (CWGAN), to generate multiple realistic, high-quality images over time based on a single input image. In the second step of growth estimation, we showed that along with classical GAN image evaluation metrics, plant-specific traits such as projected leaf area or biomass can be derived from the generated images and used for evaluation. The results for MixedCrop were compared with a dynamic process-based crop growth model. Here, the combination of data-driven crop growth models, which strongly incorporate the spatio-temporal above-ground phenotype changes, and a process-based crop growth model, which considers the theoretical plant growth knowledge, leads to a better understanding of the crop mixture dynamics. Quantitative and qualitative simulations provide a comprehensive tool to investigate how various treatments influence the above-ground phenotype of crop mixtures and their dry matter. The experiments show that the dried biomass can be estimated more accurately from predicted images the more growth influencing factors are considered, such as in our case, the field treatment or process-based simulated biomasses. In particular, the integration of process-based model output into a data-driven crop growth model is shown, which is useful to make crop growth predictions more accessible or even to re-calibrate process-based models. Incorporating all available conditions into the image generation model enables accurate estimation of plant traits in predicted (artificial) images, comparable to the accuracy achieved with real images.

Although the additional variability images show the largest uncertainties at the leaf edges, which is realistic, we see space for improvement in the uncertainty integration for long-term growth predictions. Since predictions far in the future lead to significant over-confidence in the image generation model, the weighting of the stochastic and deterministic model input should be adaptively controlled depending on the growth prediction step. In addition, the challenge of large spectral differences within an image sequence and between sites (“dataset styles”) should be addressed for better model generalizability.

## Figures and Tables

**Fig. 12:**
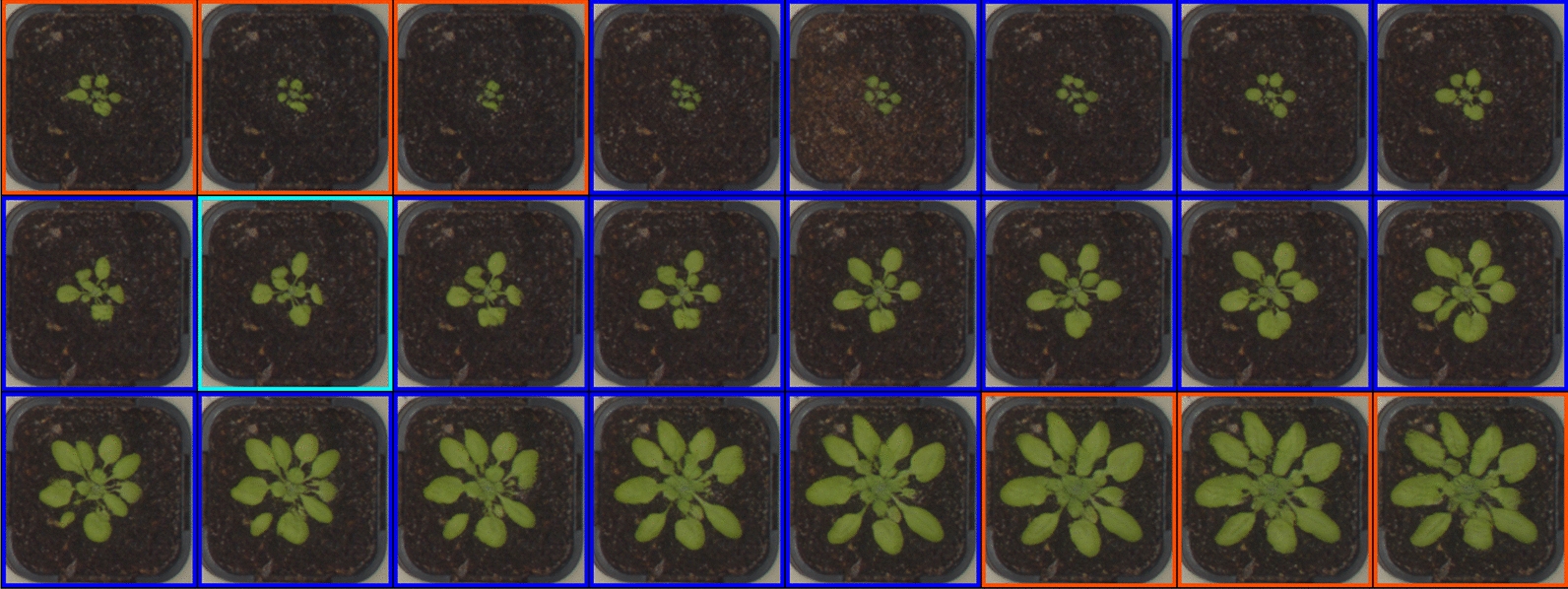
Daily Arabidopsis predictions from ${18}\,\hbox {DAS}$ to ${41}\,\hbox {DAS}$ including temporal OOD images. The input image has a cyan frame, the in-distribution images a blue frame, and the OOD images an orange frame

**Fig. 13:**
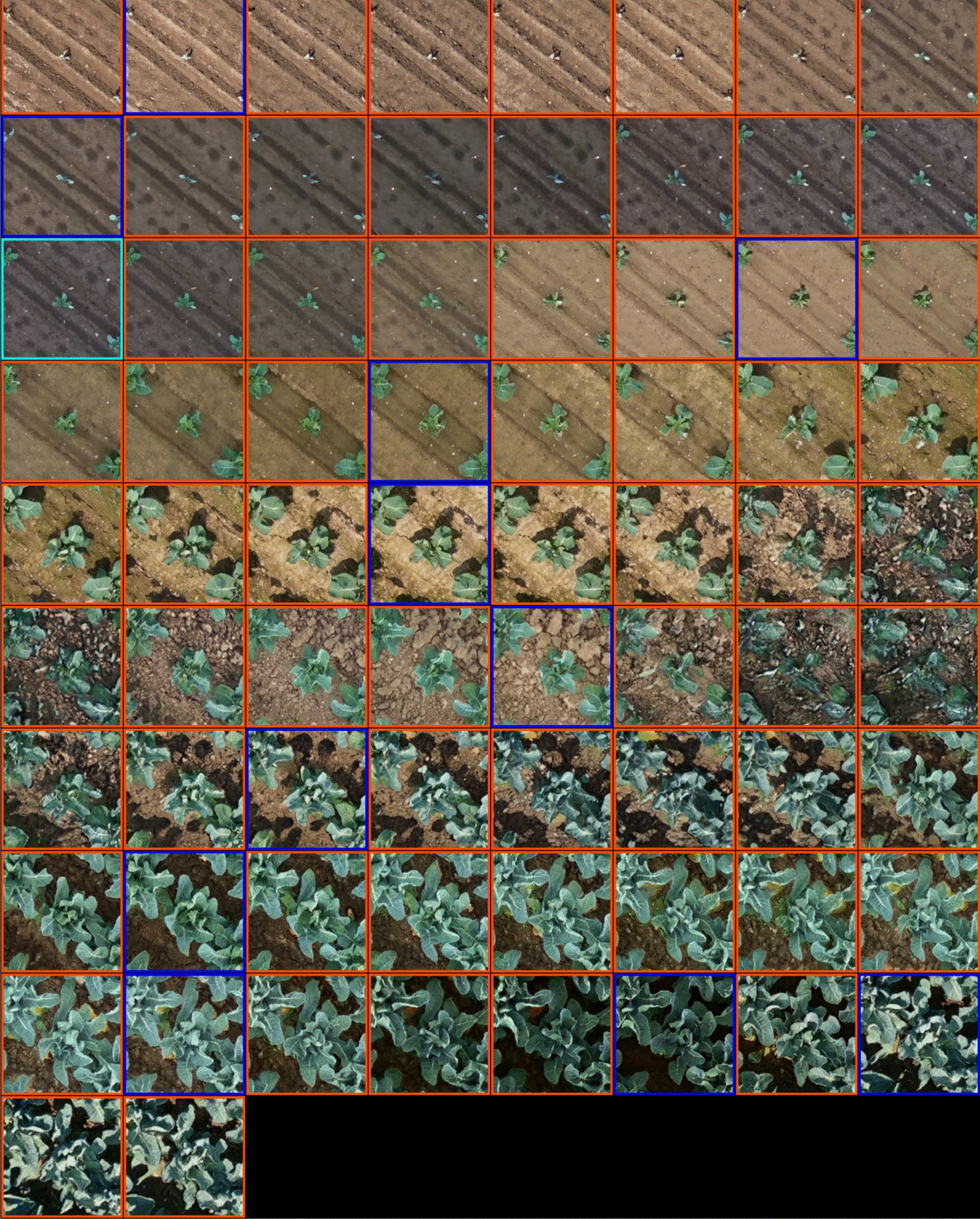
Daily GrowliFlower predictions from ${0}\,\hbox {DAP}$ to ${73}\,\hbox {DAP}$ including temporal OOD images. The input image has a cyan frame, the in-distribution images a blue frame, and the OOD images an orange frame

**Fig. 14:**
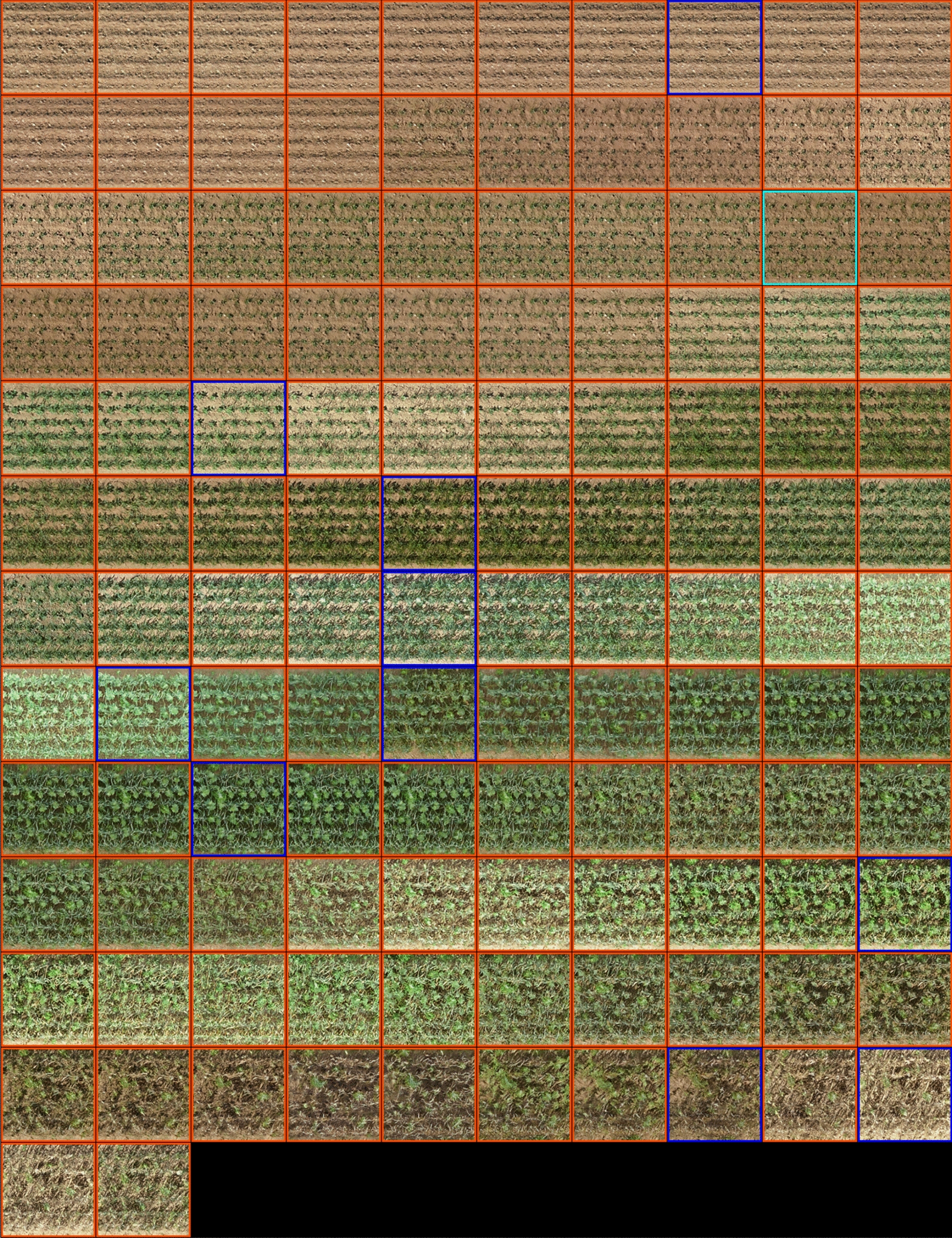
Daily Mixed-CKA predictions from ${0}\,\hbox {DAS}$ to ${121}\,\hbox {DAS}$ including temporal OOD images. The input image has a cyan frame, the in-distribution images a blue frame, and the OOD images an orange frame

**Fig. 15:**
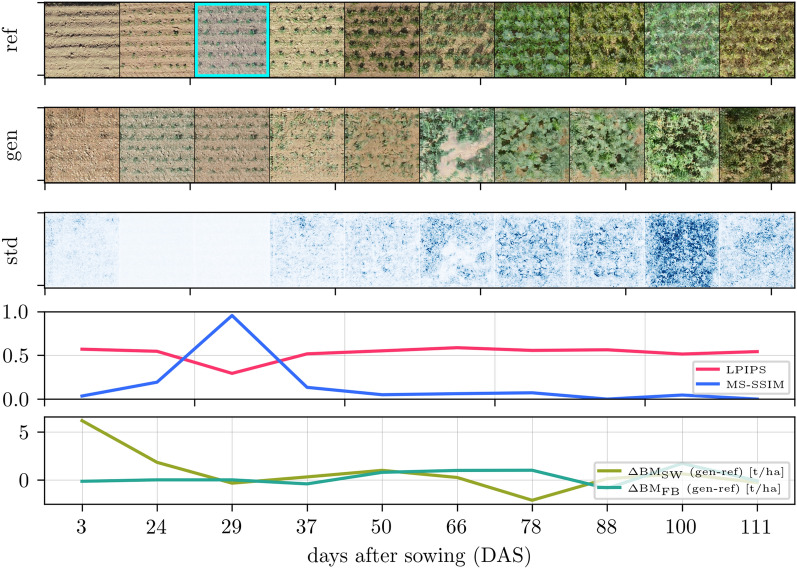
Transferability fails with predictions for Mixed-WG caused by input image ${29}\,\hbox {DAS}$ lying spectrally too far out of the training distribution (Mixed-CKA images)

**Fig. 16:**
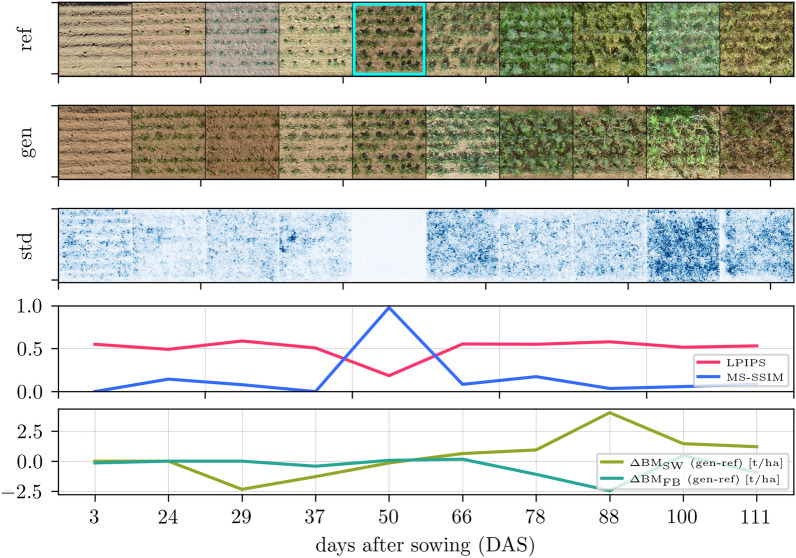
Transferability with prediction results for Mixed-WG input image ${50}\,\hbox {DAS}$ lying spectrally less far away from the ${54}\,\hbox {DAS}$-images of the training distribution (Mixed-CKA). The predicted images are qualitatively appealing, but they do not compare well with the reference because the crops of Mixed-CKA and Mixed-WG have different growth patterns

**Fig. 17:**
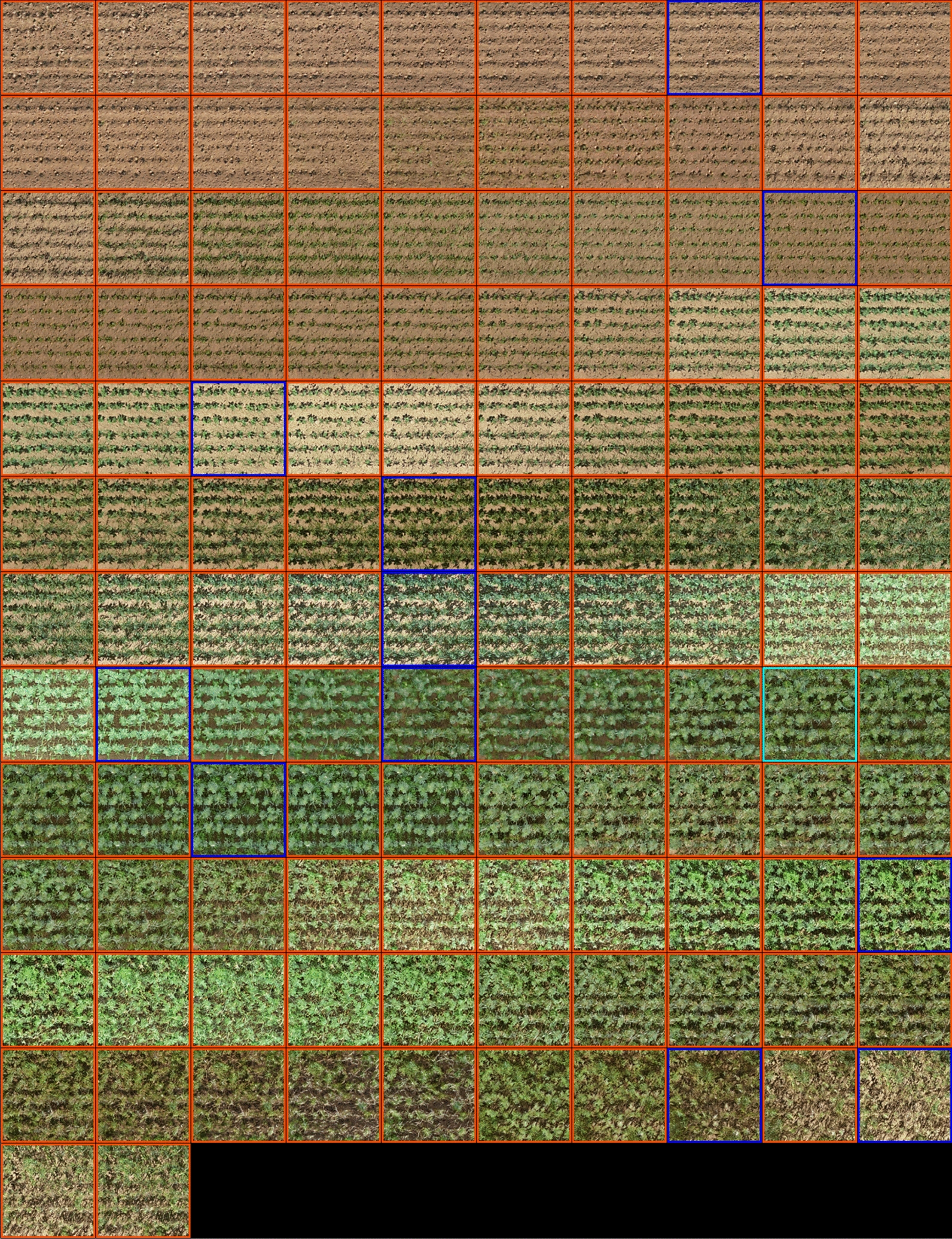
Daily Mixed-WG predictions from ${0}\,\hbox {DAS}$ to ${121}\,\hbox {DAS}$ including temporal OOD images from an image generation model trained exclusively on Mixed-CKA images

## Data Availability

Source code and links to the datasets are publicly available at https://github.com/luked12/crop-growth-cgan.

## Appendix A

**Overview of MixedCrop cultivars**

An overview of the faba bean cultivars and spring wheat entities used in the MixedCrop experiment is given in Table 7.

**Table 7:** Notation overview of species faba bean (FB) with cultivars A and B and spring wheat (SW) with cultivars 1–10 and two additional mixed groups used in this work.

FB (Faba bean)	A	Mallory
	B	Fanfare
SW (spring wheat)	1	Lennox
	2	Anabel
	3	Saludo
	4	Jasmund
	5	Sorbas
	6	Quintus
	7	KWS Starlight
	8	Chamsin
	9	Sonett
	10	SU Ahab
	11	Mix-Group 1
	12	Mix-Group 2

## Appendix B

Figs. 12, 13 and 14

**Temporal out-of-distribution predictions**

By temporal out-of-distribution (OOD), we mean images of growth stages that do not exist in the training dataset. We use the models from the respective dataset trained only on input image and time as conditions and keep the input image and the noise constant for the visualizations from the entire growth period. So we iterate over time and generate interpolations if the newly generated image lies between two training images and extrapolations if it lies temporally off the training period (early and late growth phases). The time increases by one day per image from top left to bottom right. The input image has a cyan frame, the in-distribution images a blue frame, and the OOD images an orange frame. While challenging to evaluate quantitatively because no reference images are available, consistency in the time series is readily apparent for interpolations. For extrapolations, most predictions are also realistic since plants continue to grow in the short-term extrapolated future. Notably, interpolation and extrapolation work for Mixed-WG, although the growth stage of the input image is temporally out-of-distribution. However, there are exceptions, e.g., the early growth stages of Arabidopsis are too large, and in the third row of Mixed-CKA and Mixed-WG canopy appears and vanishes again.

## Appendix C

**Spatial out-of-distribution predictions**

If the image generation model is applied to a site on whose plant image time series the model has not been trained, it is spatially out-of-distribution. Additionally, it is likely that the dataset is temporally OOD if the drone flyovers did not occur on the same days of the growing season, and thus, the time of the new input image does not exist in the training data. There, transferability fails when the spectral differences between the test image and the nearby time points in the training dataset are too large, such as ${29}\,\hbox {DAS}$ of Mixed-WG, as Fig. 15 illustrates. Some of the predicted images become blurry, and holes appear in the crop rows, which also causes the biomass estimation to give unreliable, non-usable results. Likewise, Fig. 16 demonstrates that the model can produce reasonable results despite spatio-temporal OOD, where, compared to Fig. 15, the same field patch but a different input image (21 days later) is used. Realistic results can also be achieved when not only the input image but also the images to be predicted are temporally and spatially OOD, as depicted in Fig. 17.

## Footnotes

1 Source code is publicly available: https://github.com/luked12/crop-growth-cgan

2 More information about SIMPLACE components: https://www.simplace.net/index.php/documentation
